# Selected social and lifestyle correlates of brain health markers: the Cross‐Cohort Collaboration Consortium

**DOI:** 10.1002/alz.70148

**Published:** 2025-04-10

**Authors:** Leslie Grasset, Joshua C. Bis, Stefan Frenzel, Daniel Kojis, Jeannette Simino, Amber Yaqub, Alexa Beiser, Claudine Berr, Jan Bressler, Robin Bülow, Charles S. DeCarli, Alison E. Fohner, Laura B. Harrington, Catherine Helmer, M. Arfan Ikram, Rozenn N. Lemaitre, Oscar L. Lopez, W. T. Longstreth, Julia Neitzel, Michelle C. Odden, Priya Palta, Carsten O. Schmidt, Rajesh Talluri, Meike W. Vernooij, Henry Völzke, Trudy Voortman, Quest Whalen, Katharina Wittfeld, Hans J. Grabe, Thomas H. Mosley, Bruce M. Psaty, Frank J. Wolters, Sudha Seshadri, Carole Dufouil

**Affiliations:** ^1^ University Bordeaux, Inserm Bordeaux Population Health Research Center Bordeaux France; ^2^ Cardiovascular Health Research Unit Department of Radiology & Nuclear Medicine Erasmus University of Washington Seattle Washington USA; ^3^ Department of Psychiatry and Psychotherapy University Medicine Greifswald Greifswald Germany; ^4^ The Framingham Heart Study Framingham Massachusetts USA; ^5^ Boston University School of Public Health Boston Massachusetts USA; ^6^ Department of Data Science John D. Bower School of Population Health University of Mississippi Medical Center Jackson Mississippi USA; ^7^ Memory Impairment and Neurodegenerative Dementia (MIND) Center University of Mississippi Medical Center Jackson Mississippi USA; ^8^ Department of Epidemiology Erasmus University Medical Center Rotterdam the Netherlands; ^9^ Department of Biostatistics Boston University School of Public Health Boston Massachusetts USA; ^10^ Department of Neurology Boston School of Medicine Boston Massachusetts USA; ^11^ INM, Univ Montpellier, INSERM Montpellier France; ^12^ Human Genetics Center School of Public Health University of Texas Health Science Center at Houston Houston Texas USA; ^13^ Institute of Diagnostic Radiology and Neuroradiology University Medicine Greifswald Greifswald Germany; ^14^ Department of Neurology and Center for Neuroscience University of California at Davis Sacramento California USA; ^15^ Imaging of Dementia and Aging (IDeA) Laboratory Department of Neurology University of California‐Davis Davis California USA; ^16^ Department of Epidemiology University of Washington Seattle Washington USA; ^17^ Cardiovascular Health Research Unit University of Washington Seattle Washington USA; ^18^ Kaiser Permanente Washington Health Research Institute Kaiser Permanente Washington Seattle Washington USA; ^19^ Department of Epidemiology University of Washington School of Public Health Seattle Washington USA; ^20^ Department of Health Systems Science Kaiser Permanente Bernard J. Tyson School of Medicine Pasadena California USA; ^21^ Cardiovascular Health Research Unit Department of Medicine University of Washington Seattle Washington USA; ^22^ Department of Neurology and psychiatry University of Pittsburgh Pittsburgh Pennsylvania USA; ^23^ Departments of Neurology and Epidemiology University of Washington Seattle Washington USA; ^24^ Department of Radiology and Nuclear Medicine Erasmus University Medical Center Rotterdam the Netherlands; ^25^ Department of Epidemiology Harvard TH Chan School of Public Health Boston Massachusetts USA; ^26^ Department of Epidemiology and Population Health Stanford University School of Medicine Palo Alto California USA; ^27^ Department of Neurology University of North Carolina Chapel Hill North Carolina USA; ^28^ Institute for Community Medicine University Medicine of Greifswald Greifswald Germany; ^29^ Meta‐Research Innovation Center at Stanford (METRICS) Stanford University Stanford California USA; ^30^ German Center for Neurodegenerative Disease (DZNE) partner site Rostock/Greifswald Greisfswald Germany; ^31^ Department of Medicine (Geriatrics) and Neurology University of Mississippi Medical Center Jackson Mississippi USA; ^32^ Cardiovascular Health Research Unit Departments of Medicine Epidemiology, and Health Systems and Population Health University of Washington Seattle Washington USA; ^33^ Glenn Biggs Institute for Alzheimer and Neurodegenerative Diseases The University of Texas Health Science Center at San Antonio San Antonio Texas USA; ^34^ Department of Neurology The University of Texas Health Science Center at San Antonio San Antonio Texas USA; ^35^ Pole de santé publique Centre Hospitalier Universitaire (CHU) de Bordeaux Bordeaux France

**Keywords:** cohorts, dementia, meta‐analysis, MRI, resilience

## Abstract

**INTRODUCTION:**

To investigate the associations of education level, marital status, and physical activity with dementia risk and brain MRI markers.

**METHODS:**

Data from six community‐based samples from the Cross‐Cohort Collaboration Consortium were analyzed. Self‐reported education level, marital status, and physical activity at age 60 to 75 years were harmonized. Subsamples of participants with brain MRI markers at time of exposure were selected. Associations with dementia risk and cross‐sectional MRI markers were meta‐analyzed.

**RESULTS:**

Higher education level was associated with lower dementia risk (hazard ratio [HR] = 0.65, 95% confidence interval [CI] = 0.59; 0.72 vs low level) but not significantly with brain MRI markers. Compared with being unmarried, being married was only associated with higher total brain and hippocampal volumes. Being physically active was associated with lower dementia risk (HR = 0.73, 95% CI = 0.52; 1.04), as well as larger total brain volume and smaller white matter hyperintensity volume.

**DISCUSSION:**

This study provides further evidence regarding the contribution of education level and physical activity to dementia resilience.

**Highlights:**

- Education level, marital status, and physical activity are thought to contribute to resilience against ADRD.
- We used random‐effects meta‐analysis to summarize results from six community‐based samples from the CCC.
- In this cross‐cohort meta‐analysis, higher education level and being physically active were associated with lower risk of dementia.
- In cross‐sectional analyses, being married was associated with larger TBV and HV, while being physically active was associated with larger TBV and lower WMHV.

## 1 BACKGROUND

Due to the overall increase in life expectancy worldwide, a growing part of the population is expected to be at risk for cognitive decline and age‐related brain disorders such as Alzheimer's disease and related dementias (ADRD).^1^ The augmentation of ADRD cases is likely to be the highest among the oldest old, and an important impact on overall societal costs and burden can be expected.^2^ Although some progress has recently been made regarding anti‐amyloid therapy, the clinical impact is modest with potentially important side effects. There is therefore a strong interest in identifying modifiable targets to inform future prevention strategies aimed at reducing the risk of ADRD.

Investigating factors favoring the maintenance of optimal cognition and brain health at older ages will contribute to our understanding of determinants of healthy aging. Various factors – in particular education level, physical activity, or social environment – have been shown in different studies to play a role in enhancing resilience (i.e., the ability to cope with the development of brain diseases).^3^ Indeed, higher education level, being physically active, and being married (often used as a proxy for more frequent social contact) have been shown to be associated with lower dementia risk.^4^, ^5^, ^6^, ^7^, ^8^ Yet their independent contribution still needs to be further investigated. In addition, the mechanisms involved in these associations remain uncertain. One hypothesis relates to the potential influence of social and lifestyle factors on brain structures and cerebrovascular lesions. However, the results of studies investigating these associations have been mixed. Some studies have shown less brain atrophy, cerebrovascular disease, or ADRD pathology among higher educated, physically active, or married individuals.^8^, ^9^, ^10^, ^11^, ^12^, ^13^, ^14^, ^15^, ^16^, ^17^, ^18^, ^19^ Yet other studies failed to show an association between social or lifestyle factors and structural brain markers.^11^, ^12^, ^20^, ^21^, ^22^ Efforts combining multiple cohorts in order to provide sufficient power and sample diversity with harmonized methodology are thus needed to better understand the relationships between social and lifestyle factors and cognitive and brain health.

By leveraging data from the Cross‐Cohort Collaboration Consortium (CCC), we aimed to further investigate how selected social and lifestyle factors – education level, marital status, and physical activity (assessed at age 60 to 75 years) – relate to (1) the risk of dementia over up to 15 years of follow‐up and (2) cross‐sectional brain aging markers measured at the time of factor assessment.

## 2 METHODS

### 2.1 Study population

The CCC neurology working group aims at leveraging data from existing cohort studies to clarify risk and protective factors for ADRD. This study combined data from six population‐based cohort studies, members of the CCC neurology working group: the Atherosclerosis Risk in Communities (ARIC) study, the Cardiovascular Health Study (CHS), the Framingham Heart Study (FHS), the Rotterdam Study (RS), the Study of Health in Pomerania (SHIP), and the Three‐City (3C) study.

For each study, baseline is defined as the time of assessment of factors of interest at age 60 to 75. For both aims, prevalent dementia cases at baseline were excluded from analytical samples. To study dementia risk (aim 1), participants with non‐missing factors of interest at baseline and incident dementia information over up to 15 years were included. To study brain MRI markers (aim 2), a subsample of participants with MRI brain markers available at baseline was considered. Additional information regarding analytic samples for each cohort and flow charts are presented in Supplemental Methods 1.

### 2.2 Exposures of interest

Using questionnaire data, we defined education level, marital status, and physical activity across cohorts. Education level was categorized as less than high school (0 to ≤11 years) (henceforth referred to as low education), high school/general education development (GED) degree (12 years) (intermediate), more than high school (>12 years) (high). Marital status, a proxy of social contact, was categorized as being married or in a relationship versus currently unmarried, which included being widowed, divorced, separated, or single. Physical activity was previously harmonized within the Cohorts for Heart and Aging Research in Genomic Epidemiology (CHARGE) Consortium in which most of the CCC cohorts participated; we thus decided to follow the same definition.^23^ It was dichotomized according to the following criteria. Active individuals were defined as those with ≥225 Metabolic Equivalent of Task (MET)‐min per week of moderate to vigorous leisure‐time or commuting physical activity. All other participants were considered physically inactive. When MET‐min per week measures of physical activity were not available, active individuals were defined as those engaging in at least 1 h/week of moderate‐intensity leisure‐time physical activity or commuting physical activity. In cohorts where neither MET‐min nor hours/week of physical activity were available, individuals in the highest 75% of physical activity levels of their cohort were defined as active and all other individuals as inactive. Because participants in the RS are highly active on average (more than 95% reported physical activity ≥225 MET‐min/week),^24^, ^25^ a definition based on the 75% cut‐off was chosen to define physically active participants to avoid violation of the positivity assumption despite the availability of MET‐min/week. A complete description of the assessment of the factors of interest within each cohort is presented in Methods S1.

### 2.3 Outcomes of interest

#### 2.3.1 Dementia incidence

Each cohort independently assessed dementia status over up to 15 years of follow‐up after exposures‐of‐interest measurement. Dementia cases were diagnosed based on Diagnostic and Statistical Manual of Mental Disorders, Third Edition‐Revise (DSM‐III‐R) or DSM‐IV criteria, except for SHIP. Regarding SHIP diagnoses, they were based on International Classification of Diseases, Tenth Revision (ICD‐10) codes derived from electronic health records. For individuals without incident dementia during follow‐up, censoring date was either date at last visit (for cohorts with interval censoring) or last date known to be dementia‐free (for cohorts with continuous dementia assessment) within 15 years of follow‐up.

**RESEARCH IN CONTEXT**

1. **Systematic review**: We reviewed the literature using PubMed for all articles investigating relations between resilience‐enhancing factors and dementia risk as well as brain health MRI markers published up to January 31, 2024. If selected social and lifestyle factors were often associated with dementia risk, their relations with MRI markers were less consistent.
2. **Interpretation**: In this cross‐cohort meta‐analysis, higher education level and being physically active were associated with lower risk of dementia. In cross‐sectional analyses, being married was associated with larger TBV and HV, while being physically active was associated with larger TBV and lower WMHV.
3. **Future directions**: This work suggests that selected social and lifestyle factors are of interest to promote better cognition and healthy brain aging. Additional factors such as cognitive enrichment and more detailed social network measures should also be investigated as interesting targets to enhance resilience against dementia.

#### 2.3.2 Brain imaging

For the current project, we selected the following brain MRI markers, available in most cohorts: total brain volume (TBV, in cubic centimeters [cm^3^]), total gray matter volume (GMV, in cm^3^), hippocampal volume (HV, in cm^3^), and white matter hyperintensity volume (WMHV, in cm^3^, natural log transformed). Total brain volume, GMV, HV (sum of left and right), and WMHV were determined from T1‐weighted, T2‐weighted, and fluid‐attenuated inversion recovery (FLAIR) images via automated image‐processing pipelines in all studies. Additional information regarding definitions of dementia status and brain MRI for each cohort is presented in Supplemental Methods 1.

#### 2.3.3 Covariates

Covariates assessed at the time of exposure assessment included sex, *Apolipoprotein E ε4 alele (APOE* ε4 status (zero vs one or two alleles), race/ethnicity (when available), smoking status, hypertension, diabetes, body mass index (BMI), and high depressive symptoms. Smoking status was self‐reported as current smoker versus former/never smoker. Hypertension was defined as a cut‐off of systolic/diastolic blood pressure ≥140/90 mmHg or antihypertensive medication use. Diabetes was either self‐reported or defined by medication intake or fasting glucose levels ≥126 mg/dL. High depressive symptoms were defined as a Center for Epidemiologic Studies‐Depression (CES‐D)^26^ scale score ≥16 (or ≥9 on the short CES‐D scale in ARIC).

### 2.4 Statistical analysis

To ensure harmonization of the analyses, a detailed analysis plan was shared. All analyses were conducted separately in each cohort and then meta‐analyzed. In this pre‐planned meta‐analysis, each cohort used Cox proportional hazard regression models for the outcome of dementia risk (aim 1) and linear regression models for outcomes of brain MRI markers (aim 2). Covariate selection and modeling strategy to account for potential confounders was similar for both aims, with adjustment variables selected following a Directed Acyclic Graph (DAG) approach (Figure S1). Model 1 included education level, age, sex, ethnicity (when available), and other cohort‐specific covariates such as study site or cohort. Model 2 additionally included marital status. Model 3 additionally included physical activity. Model 4 additionally included smoking status. Model 5 additionally included *APOE* ε4 status, hypertension, diabetes, BMI, and high depressive symptoms. Results from model 1 get at the main education effect, those from model 2 at main marital status effect, and those from model 3 at main physical activity effect. Results from model 4 and 5 are presented to account for factors that may be confounders, mediators, or both. Regarding aim 1, age at exposure assessment was used as time scale. Moreover, for aim 2, to account for the fact that brain MRI was often not performed at the same time as exposure assessment, with delay varying across participants and cohorts, each model was additionally adjusted for delay between exposure assessment and MRI. Each model for aim 2 also included total intracranial volume. To assess the robustness of our results, sensitivity analyses, described in Supplemental Methods 2, were undertaken.

Results from each individual cohort were combined using a random‐effects meta‐analysis, with heterogeneity across studies assessed using Cochran's Q and *I* ^2^. Cohort‐specific data were analyzed using SAS version 9.4 (FHS, ARIC, and RS) and R version 4.1.2 (CHS, 3C, SHIP). Meta‐analysis was performed with the R meta package (version 5.2‐0).^27^

## 3 RESULTS

### 3.1 Education, marital status, physical activity, and dementia risk

Overall, 16,862 participants were included in this analysis. Sample characteristics for each cohort are presented in Table 1. Mean age ranged from 66.6 (SHIP) to 71.6 (ARIC) years old, with all studies having higher proportion of females than males. Distribution of education levels differed between US (ARIC, CHS, FHS) and European (RS, SHIP, 3C) cohorts, as more than 50% of participants had more than a high school education in US cohorts, compared to around 20% in European cohorts. The proportion of participants married or in a relationship across cohorts varied from 66.8% (3C) to 80.6% (SHIP). Regarding physical activity, proportions of physically active participants varied from 55% (3C and SHIP) to 76.6% (CHS).

**TABLE 1:** Demographics and characteristics of samples with information available on social and lifestyle factors and dementia risk (*n* = 16,862).

	ARIC	CHS	FHS	RS	SHIP	3C
*N*	2673	2448	2342	2950	1962	4487
Age at lifestyle factor assessment, mean years (SD)	71.6 (1.9)	69.5 (2.5)	67.6 (3.2)	67.7 (4.1)	66.6 (4.3)	70.4 (2.6)
Male	1042 (39.0)	989 (40.4)	1091 (46.6)	1274 (43.2)	954 (48.6)	1784 (39.8)
*APOE* ε4 carriers	770 (30.3)	582 (23.8)	524 (23.0)	793 (28.7)	430 (23.5)	942 (21.0)
Education level						
<High school (low)	263 (9.8)	550 (22.5)	100 (4.3)	207 (7.0)	851 (43.4)	2688 (59.9)
High school/GED (intermediate)	905 (33.9)	735 (30.0)	673 (28.7)	2048 (69.4)	691 (35.2)	886 (19.7)
More than high school (high)	1505 (56.3)	1163 (47.5)	1569 (67.0)	695 (23.6)	420 (21.4)	913 (20.3)
Marital status						
Married/in a relationship	1869 (69.9)	1779 (72.7)	1724 (73.6)	2255 (76.4)	1581 (80.6)	2998 (66.8)
Currently unmarried	804 (30.1)	669 (27.3)	618 (26.4)	695 (23.6)	381 (19.4)	1489 (33.2)
Physically active	1734 (64.9)	1875 (76.6)	1780 (76.0)	2214 (75.1)	1077 (54.9)	2439 (54.4)
Current smoking	204 (7.7)	270 (11.0)	191 (8.2)	314 (10.7)	231 (11.8)	293 (6.5)
Diabetes	758 (28.7)	342 (14.0)	326 (13.9)	453 (15.4)	415 (21.2)	396 (8.8)
Hypertension	1878 (70.7)	1369 (55.9)	1387 (59.2)	2128 (72.2)	1425 (72.7)	3241 (72.2)
BMI, mean (SD)	29.6 (5.9)	27.0 (4.5)	28.4 (5.1)	27.6 (4.3)	29.5 (5.0)	25.8 (4.0)
High depressive symptoms	162 (6.1)	89 (3.6)	168 (8.0)	164 (5.7)	198 (10.5)	918 (20.5)
Incident dementia cases	218 (8.2)	477 (19.5)	153 (6.5)	59 (2.0)	94 (4.8)	295 (6.6)
Age at dementia diagnosis, mean (SD)	78.0 (2.1)	79.0 (3.8)	78.8 (4.8)	75.9 (3.3)	73.6 (4.2)	79.0 (3.9)

*Note*: Data are numbers (percentage) unless otherwise indicated.

Abbreviations: *APOE*, apolipoprotein E; ARIC, Atherosclerosis Risk in Communities; BMI, body mass index; CHS, Cardiovascular Health Study; FHS, Framingham Heart Study; GED, General Education Development; RS, Rotterdam Study; SHIP, Study of Health in Pomerania; 3C, Three‐City; SD, standard deviation.

US cohorts: ARIC, CHS, FHS; European cohorts: RS, SHIP, 3C.

Currently unmarried includes widowed, divorced, separated, or single.

**FIGURE 1:**
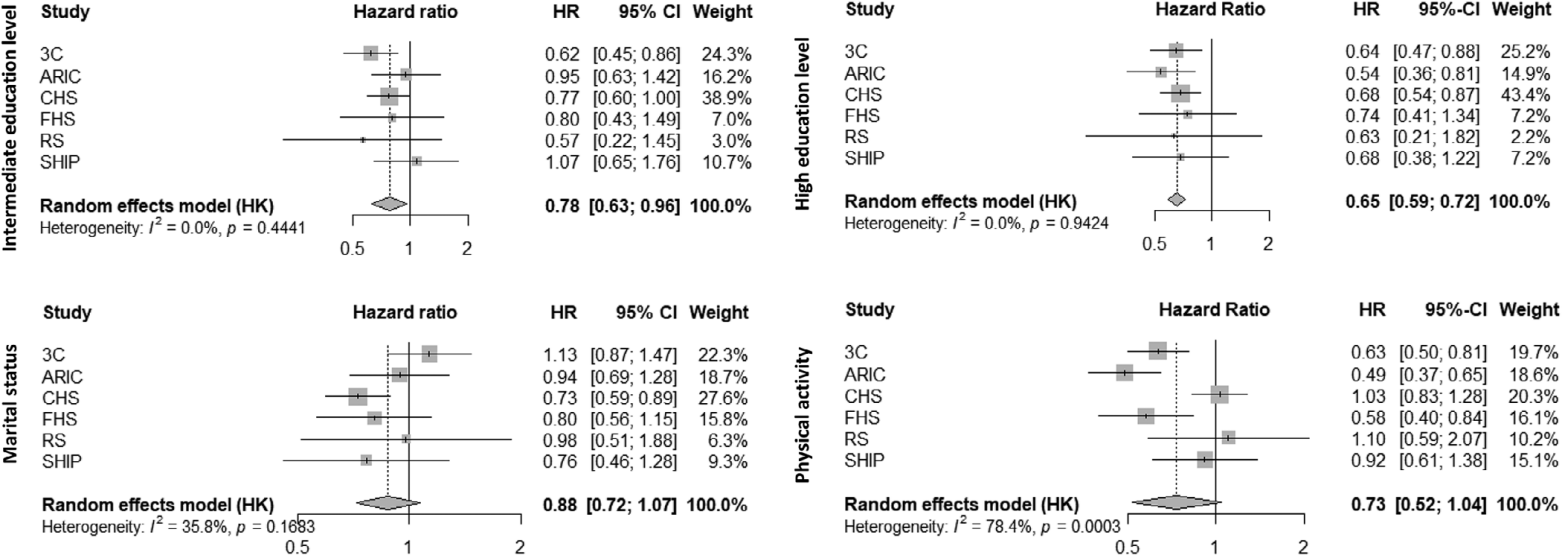
Forest plots of associations of education, marital status, and physical activity with dementia risk from main models. (Top left: intermediate vs low education level; top right: high vs low education level; bottom left: married/in a relationship vs not; bottom right: physically active vs not). ARIC: Atherosclerosis Risk in Communities, CHS: Cardiovascular Health Study, FHS: Framingham Heart Study, RS: Rotterdam Study, SHIP: Study of Health in Pomerania, 3C: Three‐city. Individual (square) and overall (diamond) coefficients are displayed. Models for education were adjusted for age, sex, ethnicity (when applicable), and other cohort‐specific covariates. Models for marital status were adjusted for age, sex, education level, ethnicity (when applicable), and other cohort‐specific covariates. Models for physical activity were adjusted for age, sex, education level, marital status, ethnicity (when applicable), and other cohort‐specific covariates.

Meta‐analyzed estimates of the associations of education, marital status, and physical activity with the risk of dementia are displayed in Figure 1 and Table S1 for main and additionally adjusted models, respectively. In the meta‐analysis, intermediate (HR_M1 _= 0.78, 95% CI = 0.63; 0.96) and high education levels (HR_M1 _= 0.65, 95% CI = 0.59; 0.72) compared with low education were statistically significantly associated with lower risks of dementia. There was also a trend toward a lower risk of dementia for physical activity compared to inactivity (HR_M3 _= 0.73, 95% CI = 0.52; 1.04). Marital status was not statistically significantly associated with dementia risk in either of the models (HR_M2_ = 0.88, 95% CI = 0.72; 1.07). While almost no heterogeneity was found for associations between education levels (*I* ^2^ = 0%) and dementia risk, there was substantial heterogeneity for marital status (*I* ^2^ = 35.8%) and physical activity (*I* ^2^ = 78.4%). Analyses from additionally adjusted models led to similar results.

After exclusion of the estimate from the RS for physical activity, the meta‐analyzed association between physical activity and dementia risk was similar (Table S2). The associations between education levels and risk of dementia stratified by geographical location (European Union vs USA) are presented in Table S3. This sensitivity analysis did not show significant geographical differences. In addition, although the magnitude of the associations between education levels and dementia risk varied across definitions within the 3C study, higher education levels were consistently associated with lower risk of dementia (Table S4).

### 3.2 Education, marital status, physical activity, and brain MRI markers

For this analysis, 8517 participants with available information on education, marital status, physical activity, and brain MRI markers made up the analytical sample. All cohorts included more females than males, with the mean age ranging from 66.3 (SHIP) to 71.6 (ARIC) years old (Table S5). Distribution of education levels across US/European cohorts were similar to the dementia analysis. The proportion of participants married or in a relationship varied from 63.8% to 83.2% across cohorts, and the proportion of physically active participants ranged from 54.8% to 77.8%.

Meta‐analyzed estimates of the associations between social and lifestyle factors with brain MRI markers are presented in Table 2 for the main models and in Table S6 for additionally adjusted models. Overall, compared with being currently unmarried, being married was significantly associated with larger TBV (from model 2 mean difference: 2.48 cm^3^, 95% CI = 0.76; 4.20), GMV (1.83 cm^3^, 95% CI = 0.22; 3.45), and HV (0.04 cm^3^, 95% CI = 0.01; 0.06). Compared with physically inactive participants, physically active participants had significantly larger TBV (from model 3 mean difference: 5.02 cm^3^, 95% CI = 2.71; 7.34) and GMV (3.19 cm^3^, 95% CI = 0.60; 5.78) and lower WMHV (−0.08, 95% CI = −0.15; −0.01). There was no significant association between intermediate education level and brain MRI markers in either of the models and only non‐significant trends between high education level and brain volumes compared to low education (from model 1 TBV mean difference = 2.96 cm^3^, 95% CI = −0.73; 6.64). Heterogeneity varied from *I* ^2^ = 0.00 to 44% and was higher for high education level and physical activity. Results were similar in additionally adjusted models, with only slightly attenuated estimates, except for marital status and GMV where associations were no longer significant.

**TABLE 2:** Overall associations between education, marital status, physical activity, and brain MRI markers in main models.

				Heterogeneity test		
Outcome	Exposure	No. studies	Overall effect (95% CI)	*Q*	*p* value	*I^2^*
**TBV**	Intermediate education	5	0.677 (−3.144; 4.499)	3.59	0.465	0.0
	High education	5	2.955 (−0.731; 6.642)	3.17	0.529	0.0
	Married	5	2.483 (0.762; 4.204)	1.70	0.791	0.0
	Physically active	5	5.021 (2.707; 7.335)	3.31	0.508	0.0
**GMV**	Intermediate education	5	0.071 (−2.613; 2.755)	3.36	0.500	0.0
	High education	5	2.645 (−1.245; 6.534)	7.12	0.130	43.8
	Married	5	1.834 (0.222; 3.445)	2.65	0.619	0.0
	Physically active	5	3.188 (0.595; 5.781)	6.74	0.150	40.7
**HV**	Intermediate education	5	−0.019 (−0.096; 0.057)	4.82	0.307	17.0
	High education	5	0.036 (−0.038; 0.110)	4.62	0.329	13.4
	Married	5	0.036 (0.014; 0.059)	1.00	0.910	0.0
	Physically active	5	0.026 (−0.027; 0.078)	5.50	0.239	27.3
**WMHV**	Intermediate education	6	−0.020 (−0.055; 0.015)	2.12	0.832	0.0
	High education	6	−0.021 (−0.072; 0.030)	3.97	0.554	0.0
	Married	6	−0.038 (−0.099; 0.023)	7.34	0.197	31.9
	Physically active	6	−0.081 (−0.148; −0.014)	8.84	0.116	43.5

*Note*: Intermediate and higher education versus low education, married or in a relationship versus currently unmarried, physically active versus inactive.

Brain volumes unit is cm^3^ and WMHV were log‐transformed. Models for education were adjusted for age, sex, ethnicity (when applicable), and other cohort specific covariates. Models for marital status were adjusted for age, sex, education level, ethnicity (when applicable), and other cohort‐specific covariates. Models for physical activity were adjusted for age, sex, education level, marital status, ethnicity (when applicable), and other cohort‐specific covariates. Heterogeneity measures: Cochran's Q statistic and associated *p* value, *I^2^*: percentage of variation across studies due to heterogeneity rather than chance.

Abbreviations: GMV, total gray matter volume; HV, hippocampal volume; TBV, total brain volume; WMHV, white matter hyperintensity volume.

A sensitivity analysis with weighted models accounting for selection into the MRI sample produced similar results, with somewhat stronger estimates (Table S7). Then, excluding graded measure of white matter lesions from CHS or physical activity estimates from RS yielded similar conclusions (Table S2 and S8, respectively). In addition, we did not find evidence of significant geographical differences between education levels and brain MRI markers (Table S3).

## 4 DISCUSSION

In this meta‐analysis combining data from six population‐based cohort studies, we showed in a sample of 16,862 older adults that higher education levels and physical activity versus inactivity at age 60 to 75 years were associated with lower risk of subsequent dementia over a period up to 15 years. In addition, being married was significantly associated with higher TBV and HV than being currently unmarried, while being physically active was significantly associated with larger TBV and GMV and lower WMHV than inactive. We showed a non‐significant trend toward larger brain volume with high education (vs low) and no statistically significant association between marital status and dementia risk. Accounting for *APOE* ε4 genotype or excluding *APOE* ε2 carriers did not modify our conclusions (data not shown). Additional investigations, such as testing for effect modification, are required to determine genetic influences on modifiable risk factor effect.

Increasing attention has been given to social and lifestyle factors with regard to healthy brain aging, as they represent attractive targets for prevention strategies.^3^ Regarding education level, this work supports evidence that suggests higher education enhances resilience against ADRD, with participants with high and, to a lesser extent, intermediate education levels presenting lower dementia risk compared with individuals with low education level.^4^, ^28^, ^29^ Higher education levels likely delay ADRD onset by increasing cognitive reserve, improving the adaptability of the brain in the face of life‐course‐related brain changes and brain injury.^30^ Studies showing associations between education level and changes in functional brain network further support this possibility.^31^ Moreover, although non‐statistically significant, our results seem to show a trend toward larger brain volumes for high versus low education level, but not with WMHV and not for intermediate education level. Other studies have reported education‐related differences in brain structure.^32^, ^33^ These observations may be consistent with the brain reserve hypothesis, suggesting that high education enhances the neurobiological status of the brain,^30^ and with studies showing no associations between education level and markers of brain pathology.^4^, ^12^, ^21^ The lack of significant association of education level with MRI markers may be due to the heterogeneity of results between cohorts, likely due to differences in underlying education systems and in definitions of the exposure variable. Finally, with regard to the effect size of education associations compared to the other exposure effect sizes, power issues may also be an explanation.

Physical activity has often been linked to global and cardiovascular health, yet its independent association with cognitive and brain health still needs to be validated. Our results are consistent with different studies showing an association between physical activity and a lower dementia risk overall.^34^, ^35^, ^36^ Moreover, our results are consistent with others showing higher brain volumes and lower white matter lesions in physically active individuals.^37^, ^38^, ^39^, ^40^, ^41^, ^42^ Yet some studies have not found evidence of associations between physical activity and brain structures and pathologies.^20^, ^22^, ^43^, ^44^ For example, another study using a similar meta‐analytic approach showed no associations between physical activity and GMV or HV, which the authors attributed to a potential bias due to the extremely low prevalence of physical inactivity in their sample.^45^ Differences in conclusions across studies may indeed be due to heterogeneity in physical activity assessment (e.g., self‐reported vs objectively measured), in physical activity levels of the samples, and in samples’ characteristics. Our work suggests that physical activity may be related to healthy cognitive aging through improved brain health. Physical activity may indicate a globally healthier lifestyle, with physically active individuals presenting fewer cardiovascular risk factors, thereby lowering global cerebral atrophy and the development of cerebrovascular lesions.^46^ The neuroprotective effects of physical activity in the brain could be explained by different underlying mechanisms such as increased cerebral blood flow, increased neurogenesis and neurotrophic factors, or lower oxidative stress.^47^ In addition, physical activity has also been shown to be a contributing factor to cognitive reserve, mitigating the negative effect of cerebrovascular or AD pathologies on cognitive decline and dementia risk.^22^ Yet, it is important to note that ADRD may lead to decreased physical engagement, and reverse causation cannot be excluded.^48^

Finally, frequent social contacts have been shown to be associated with healthier aging and reduced ADRD risk.^5^, ^49^ At older ages, many people are widowed or divorced, likely reducing daily social interactions. Marital status is thus an important contributor to social health in these populations. It could either enhance cognitive reserve or reduce harmful lifestyle behaviors.^50^, ^51^ However in this work, we did not observe a statistically significant association between being married and dementia risk. This may be attributed to high heterogeneity in results across cohorts, adjustment for physical activity, or the fact that the different categories (i.e., widowed, divorced, or lifelong single) seem to show differences in associations with dementia risk compared to married individuals.^7^ Moreover, a few studies have shown that it is the quality of interactions, rather than the quantity, that protects older adults from dementia, which could not be measured in this study.^52^, ^53^ Finally, differences by gender may also modify the association between marriage and health outcomes. Yet we found that being married was associated with higher TBV and HV, which has also been shown in a few other studies.^9^, ^54^ Additional studies using extensive information on social factors and longitudinal measures of brain and cognitive health would help to disentangle the contribution of social environment to healthy aging.

Our study has some limitations. First, although harmonized across studies, social and lifestyle factors may differ across countries. Indeed, there seem to be clear differences in the partition of education levels in US cohorts compared to European cohorts, probably due to differences in educational systems, but perhaps also to differences in the exposure definition or to the level of education of the populations recruited across cohorts. Yet sensitivity analyses exploring the effect of contextual differences as well as differences in education level definitions tended to confirm the main conclusions of this work. In addition, there were differences in physical activity assessments used in the participating cohorts. Thus, due to the unavailability of continuous measures in some cohorts, physical activity was dichotomized into inactive versus active individuals, which resulted in a loss of information and power. Moreover, there may also be differences in physical activity levels across cohorts, such as highly active participants in the RS. Second, due to the cross‐sectional nature of MRI analysis, causal inference should be made with caution. Social and lifestyle factors were self‐reported; thus, measurement bias and reverse causality cannot be excluded for both dementia and MRI analyses. Indeed, physical inactivity in older ages might also occur as part of the dementia prodrome. Finally, although dementia diagnosis procedures and volumetric image analysis procedures of brain MRI may present some differences across cohorts, prior work combining data from these cohorts has reported robust results and support our meta‐analytic approach.

Despite these limitations, this study has important strengths and contributes to the literature on the influences of social and lifestyle factors on cognitive and brain aging. Leveraging data from six cohorts provided a large sample to detect associations between these factors and markers of cognition and brain health. Moreover, variable definitions as well as study design were harmonized across studies to limit heterogeneity.

To conclude, we showed that higher education levels and physical activity were associated with lower risk of dementia, while being married and physically active were significantly associated with higher brain volumes and lower burden of white matter hyperintensities. Although further studies are needed to fully comprehend the contribution of selected social and lifestyle factors to resilience mechanisms, this work provides additional understanding of their associations with healthy cognitive and brain aging.

## CONFLICT OF INTEREST STATEMENT

Psaty serves on the Steering Committee of the Yale Open Data Access Project funded by Johnson & Johnson. Grabe has received travel grants and speaker honoraria from Neuraxpharm, Servier, Indorsia, and Janssen Cilag. DeCarli has received consulting fees from Eisai and Novo Nordisk pharmaceuticals. Author disclosures are available in the supporting information.

## CONSENT STATEMENT

All studies were approved by corresponding ethics committees. All participants signed written informed consent.

## Supporting information



Supporting Information

Supporting Information
