# Supplementary material for: Selected social and lifestyle correlates of brain health markers: the Cross‐Cohort Collaboration Consortium
Source: Alzheimers Dement. 2025 Apr 10;21(4):e70148. doi: 10.1002/alz.70148 (PMC11982914; doi:10.1002/alz.70148)
Supplement: Supplementary file 1 — Supporting Information [file ALZ-21-e70148-s001.docx]

**Supplementary materials**

**Supplemental Methods 1:** Cohorts information

**Supplemental Methods 2:** Description of sensitivity analyses

**Supplemental Figure 1:** Directed Acyclic Graphs (DAGs) of the associations between education level, marital status, or physical activity and brain health outcomes

**Supplemental Table 1:** Overall associations between education, marital status, physical activity and dementia risk across models.

**Supplemental Table 2:** Meta-analyzed associations between physical activity (vs. inactivity) and dementia and MRI outcomes after exclusion of the Rotterdam Study.

**Supplemental Table 3:** Geographical differences in the associations of education levels and outcomes of interest

**Supplemental Table 4:** Associations between different definition cut-offs of education level and dementia risk in the 3C study

**Supplemental Table 5:** Demographics and characteristics of samples with information available on social and lifestyle factors and MRI markers (n=8517)

**Supplemental Table 6:** Overall associations between education, marital status, physical activity and brain MRI markers across models.

**Supplemental Table 7:** Associations between exposures of interest and brain MRI markers, in the original and weighted models, in the 3C study.

**Supplemental Table 8:** Meta-analyzed associations between exposures of interest and white matter hyperintensities volume after excluding the Cardiovascular Health Study

**Supplemental Methods 1: Cohorts information**

**The Three City (3C) Study**

**General Information**

The 3C Study is conducted in three areas of France: Bordeaux (South-West), Dijon (North-East) and Montpellier (South-East). Details of this study have previously been published.(1) To be eligible for recruitment into the study, persons had to be 1/living in these cities or their suburbs and registered on the electoral rolls, 2/aged 65 years and over, and 3/not institutionalized. Twenty-four percent of the eligible persons selected on the electoral rolls (n = 34,922) could not be reached; among those contacted, the acceptance rate was 37%. A total of 9,294 persons were included: 2104 from Bordeaux, 4931 from Dijon, and 2259 from Montpellier. Baseline examinations took place between March 1999 and March 2001. Participants were then followed over at least 12 years, every 2 or 3 years. Repeated cognitive evaluations, as well as active assessment of dementia cases have been realized at each follow-up. This research adhered to the principles of the Declaration of Helsinki. The ethics committee of the Kremlin-Bicêtre University Hospital and Sud-Méditerranée III (France) approved the 3C study protocol. All participants gave written informed consent.

**Data Collection**

At each study wave, a standardized questionnaire assessing socio-demographic, medical, cognitive, and functional characteristics was administered at home by trained neuropsychologists during face-to-face interviews. Ninety percent of the participants accepted to have blood sampling for the measurement of biological parameters at baseline. At baseline, MRI scans were proposed to participants aged ≤80 years. A total of 2,554 MRI examinations have been performed. Type 2 diabetes was defined as a fasting serum glucose level ≥7.0 mmol/L (126 mg/dL), a nonfasting serum glucose level ≥11.1 mmol/L (200 mg/dL), and/or antidiabetic drug intake. Hypertension was defined by either measured systolic blood pressure ≥ 140 mmHg or diastolic blood pressure ≥ 90 mmHg, antihypertensive drug intake. Depressive symptomatology was assessed with the Center for Epidemiological Studies-Depression (CESD) scale, using scores of > 16 as indicators of a clinically relevant level of depressive symptoms. APOE-ε4 status was defined as at least one ε4 allele carried versus none.(2)

*Social/Lifestyle factors –* Education level, marital status, and physical activity were self-reported at baseline. For Dijon and Montpellier, participants were reporting the frequency at which they are practicing a physical activity. Physically active participants reported practicing a physical activity sometimes, regularly or often. For Bordeaux, participants were asked whether they were practicing a physical activity, and provided the number of hours of practice per week. To standardized physical activity distribution, anyone answering yes was considered physically active.

*Dementia Ascertainment –* Dementia diagnosis was realized using a standardized three-step procedure.(1) The first step was a cognitive evaluation by trained neuropsychologists using a series of psychometric tests. Participants who were suspected of dementia, based on their neuropsychological performance or decline relative to a previous examination were then examined for further medical assessments. Finally, each case was discussed by a validation committee composed of neurologists and geriatricians to establish diagnosis and classify etiology. The diagnosis of dementia was based on the Diagnostic and Statistical Manual of Mental Disorders - Fourth Edition (DSM-IV) criteria. Dementia subtyping was based on the National Institute of Neurological and Communicative Disorders and Stroke–Alzheimer’s Disease and Related Disorders Association (NINDS-ADRDA) criteria for AD, and on the National Institute of Neurological Disorders and Stroke–Association Internationale pour la Recherche et l’Enseignement en Neurosciences (NINDS-AIREN) criteria for vascular dementia. Mixed dementia was defined as a diagnosis of AD with either cerebrovascular lesions on brain imaging when available or a documented history of stroke and the presence of prominent executive function deficits in addition to an AD-type cognitive profile.

*Magnetic Resonance Imaging* – The protocol for cranial MRI, using either a 1.5-T Magnetom (Siemens, Erlangen, Germany) in Dijon or an 1.5T Gyroscan Intera system (Philips Medical Systems, Netherlands) in Bordeaux, has been described in detail previously.(3) Using voxel-based morphometry techniques, total intracranial volume (TIV) was computed by summing grey matter, white matter, and cerebro-spinal fluid (CSF) volumes. Hippocampal volume was defined as the sum of left and right hemisphere volume.(4) A fully automatic image processing software was developed to detect and quantify white matter hyperintensities (WMH).(3) WMH volume was calculated by summing the volumes of all the lesions detected. WMH were detected in every participant. Cortical brain infarct (CBI) of presumed vascular origin were visually rated on T1-, T2-, and proton density-weighted images in Dijon only. Characteristics of lesions were visualized simultaneously in axial, coronal, and sagittal planes. They were defined as focal lesions 3 to 15 mm in diameter with the same signal characteristics as CSF on all sequences, located in basal ganglia, brainstem, or cerebral white matter.(5)

**Analytic Sample**

Of 9078 dementia-free participants at baseline in the 3C study, 4837 had available information regarding exposures of interest between age 60 and 75 years at baseline. For the dementia risk aim, 350 participants were excluded from the sample due to lack of dementia follow-up after exposures of interest measurement, thus the final analytic sample included 4487 participants. For the MRI markers aim, 3245 participants were excluded as they did not have MRI assessment and 68 participants were further excluded due to missing total intracranial volume information, thus the final analytic sample included 1524 participants.


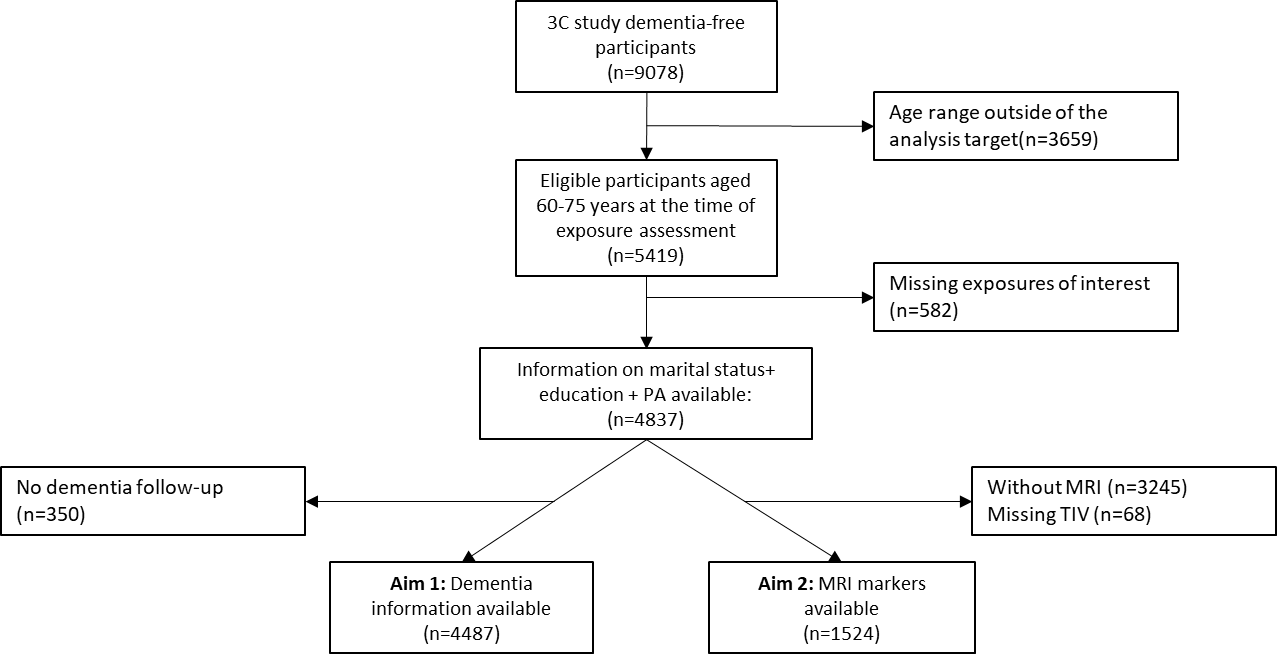


**Funding**

The 3C study is conducted under a partnership agreement between the Institut National de la Santé et de la Recherche Médicale (INSERM), the Victor Segalen-Bordeaux II University, and Sanofi-Aventis. The Fondation pour la Recherche Médicale funded the preparation and initiation of the study. The Fondation Plan Alzheimer partly funded the follow-up of the study. The 3C study is also supported by the Caisse Nationale Maladie des Travailleurs Salariés, Direction Générale de la Santé, Mutuelle Générale de l’Education Nationale, Institut de la Longévité, Conseils Régionaux of Aquitaine and Bourgogne, Fondation de France, la Caisse Nationale de Solidarité et d’Autonomie, and the Ministry of Research-INSERM Programme Cohortes et collections de données biologiques.

**References**

1. The 3C Study Group. Vascular factors and risk of dementia: design of the Three-City Study and baseline characteristics of the study population. Neuroepidemiology. 2003;22(6):316-25.

2. Dufouil C, Richard F, Fievet N, Dartigues JF, Ritchie K, Tzourio C, et al. APOE genotype, cholesterol level, lipid-lowering treatment, and dementia: the Three-City Study. Neurology. 2005;64(9):1531-8.

3. Maillard P, Delcroix N, Crivello F, Dufouil C, Gicquel S, Joliot M, et al. An automated procedure for the assessment of white matter hyperintensities by multispectral (T1, T2, PD) MRI and an evaluation of its between-centre reproducibility based on two large community databases. Neuroradiology. 2008;50(1):31-42.

4. Crivello F, Lemaitre H, Dufouil C, Grassiot B, Delcroix N, Tzourio-Mazoyer N, et al. Effects of ApoE-epsilon4 allele load and age on the rates of grey matter and hippocampal volumes loss in a longitudinal cohort of 1186 healthy elderly persons. Neuroimage. 2010;53(3):1064-9.

5. Satizabal CL, Zhu YC, Mazoyer B, Dufouil C, Tzourio C. Circulating IL-6 and CRP are associated with MRI findings in the elderly: the 3C-Dijon Study. Neurology. 2012;78(10):720-7.

6. Fletcher E, Singh B, Harvey D, Carmichael O, Decarli C. Adaptive image segmentation for robust measurement of longitudinal brain tissue change. Conf Proc IEEE Eng Med Biol Soc. 2012;2012:5319-22.

7. Maillard P, Lu H, Arfanakis K, Gold BT, Bauer CE, Zachariou V, et al. Instrumental validation of free water, peak-width of skeletonized mean diffusivity, and white matter hyperintensities: MarkVCID neuroimaging kits. Alzheimers Dement (Amst). 2022;14(1):e12261.

8. Fletcher E, Carmichael O, Decarli C. MRI non-uniformity correction through interleaved bias estimation and B-spline deformation with a template. Conf Proc IEEE Eng Med Biol Soc. 2012;2012:106-9.

9. Rueckert D, Aljabar P, Heckemann RA, Hajnal JV, Hammers A. Diffeomorphic registration using B-splines. Med Image Comput Comput Assist Interv. 2006;9(Pt 2):702-9.

10. Aljabar P, Heckemann RA, Hammers A, Hajnal JV, Rueckert D. Multi-atlas based segmentation of brain images: atlas selection and its effect on accuracy. Neuroimage. 2009;46(3):726-38.

11. Wardlaw JM, Smith EE, Biessels GJ, Cordonnier C, Fazekas F, Frayne R, et al. Neuroimaging standards for research into small vessel disease and its contribution to ageing and neurodegeneration. Lancet Neurol. 2013;12(8):822-38.

12. DeCarli C, Massaro J, Harvey D, Hald J, Tullberg M, Au R, et al. Measures of brain morphology and infarction in the framingham heart study: establishing what is normal. Neurobiol Aging. 2005;26(4):491-510.

**The Atherosclerosis Risk in Communities (ARIC) Study**

**General Information**

The Atherosclerosis Risk in Communities (ARIC) study is a prospective epidemiologic investigation of the causes of atherosclerosis, its clinical consequences, and differences in cardiovascular risk factors, disease, and medical care by geography, race, sex, and time [1]. Initiated in 1987 with financial support from the National Heart, Lung, and Blood Institute (NHLBI), the study recruited 15,792 middle-aged (45-64 years) participants from four U.S. communities: Forsyth County, North Carolina; Jackson, Mississippi; suburban Minneapolis, Minnesota; and Washington County, Maryland [1]. The suburban Minneapolis, Washington County, and Forsyth participants represented the racial/ethnic mix of their community, with the former two comprised of White participants and the latter comprised of both Black and White participants. The Jackson field center recruited Black participants only.

To evaluate social and lifestyle factor associations with incident dementia, this investigation used Black and White participants from the fifth examination (2011-2013). To assess social and lifestyle factor associations with brain volumes, this investigation used a subset of the fifth examination participants who underwent brain magnetic resonance imaging as part of the ARIC Neurocognitive study [2]. Participants considered for MRI scans included (1) all participants with a previous brain MRI from an ancillary study conducted in 2004-2006; 2) all individuals with cognitive impairment as evidenced by low Mini-Mental State Examination scores (<19 for Blacks and <21 for Whites) at the fifth clinic visit or low age-, race-, and education-adjusted z scores on at least 1 of 5 cognitive domains (failure on the clock reading test for visuospatial domain or z scores below −1.5 for memory, language, executive function, or attention domains) at the fifth visit accompanied by cognitive decline from prior visits (below the 10th percentile for change in the digit symbol substitution, delayed word recall, or word fluency test or below the 20th percentile for change on ≥2 of these tests); and (3) an age- and field center-stratified random sample of cognitively normal individuals.

**Data Collection**

Demographic variables (sex, race, and date of birth) were self-reported at the baseline visit (1987-1989). Body-mass-index was calculated from the weight and standing height measured at the fifth examination, while smoking status was obtained via self-report at the fifth visit. Diabetes was determined from blood drawn or medication use reported at the fifth clinic visit; participants were classified as diabetic if they had fasting blood glucose values ≥ 126 mg/dL, non-fasting glucose values ≥200 mg/dL, or used oral medication or insulin for diabetes. Hypertension was determined from the average of the second and third readings of systolic and diastolic blood pressure at the fifth clinic exam. Participants were labeled hypertensive if they had an average systolic blood pressure ≥ 140 mmHg, diastolic blood pressure ≥ 90 mmHg, or used medication for high blood pressure. APOE genotypes were determined using TaqMan assays and the ABI 7700 Sequence Detection System (Applied Biosystems, Foster City, CA). APOE ε4 carrier status (0 = No, 1 = Yes) indicated whether a participant carried at least one copy of the ε4 allele. The Center for Epidemiologic Studies Depression (CES-D) Scale 11-item questionnaire was used at the fifth visit [14]. This version of the CESD is highly correlated with the original 20-item version and is less taxing for the older participants [15]. Participants with CESD scores ≥ 9 were considered to have high depressive symptoms [14][16].

*Social/Lifestyle factors –* Education was ascertained from an interview at the first examination (1987-1989). Participants were asked the highest grade or year of school completed, including trade/vocational school or college. For this investigation, the response was categorized as less than high school (<12 years), high school diploma (12 years) or general education development (GED) credential, or more than high school (any amount of vocational training or college).

Marital status was ascertained during annual telephone interviews. The marital status was extracted from the annual interview closest to and within one year of the fifth examination date. Participants were asked whether they were married, widowed, divorced, separated, or never married. For this investigation, the response was categorized as married versus not married (widowed, divorced, separated, or never married).

Physical activity was determined from a modified version of the Baecke Physical Activity Questionnaire administered by an interviewer at the fifth examination [10]. Participants reported up to four exercise or sports activities most frequently done, including duration and frequency [11]. Using the 2011 Compendium of Physical Activities [12], each activity type was assigned a metabolic equivalent of task (MET) from 1 to 12. The multiplication of the MET value, frequency, and duration yielded a MET-minutes per week for each activity reported [13]. Moderate-to-vigorous intensity leisure-time physical activity was aggregated across all activity types where MET ≥ 3. Participants who reported no sports or leisure -time activities were given a value of 0 MET-minutes per week. Inactive individuals were defined as those with less than 225 MET-minutes per week of moderate-to-vigorous leisure-time physical activity. All other participants were deemed physically active.

*Dementia Ascertainment –* Dementia status was available through the latest date of dementia surveillance (November 15, 2019) or censoring because of death. Dementia determination in ARIC is extensive with multiple sources of information [17-18]. An in-person comprehensive neuropsychological battery was administered to participants at visits 5 through 7 and an informant interview was conducted in a subset of participants [17]. A diagnosis of dementia was assigned by applying a computer diagnostic algorithm to test results and obtaining expert adjudication based on the Diagnostic and Statistical Manual of Mental Disorders (Fifth Edition) and the criteria outlined by the National Institutes of Health/ National Institute on Aging [19]. In addition to dementia diagnoses determined in person, dementia was also determined by participant telephone cognitive testing as well as informant interview as previously described [18]. Additional dementia cases were identified through hospital discharge codes (International Classification of Diseases, Ninth Revision codes 331.0, 290.4, 290.0, 290.1, 290.2, 290.3, 290.9, 294.1, 294.2, 294.8, 294.9, 331.1, 331.2, 331.8, and 331.9) or death certificates [17]. Diagnoses were prioritized with the reviewer diagnosis being given the highest priority, followed by the algorithmic syndromic diagnosis, the AD8 Dementia Screening Interview, the Six Item Screener (SIS), hospitalization dementia discharge codes, and the death certificate. For those diagnosed with dementia, one of the following dates were available: date of the earliest hospitalization with a dementia code, date of death with a dementia code, the clinic visit date when dementia was observed, or the telephone interview, AD8, or SIS dates. The date of incident dementia was given a 6-month lead time recognizing that the true onset was likely before the date listed.

*Magnetic Resonance Imaging* – The MRI scans were performed using 3T scanners at each study site (Maryland: Siemens Verio; North Carolina: Siemens Skyra; Minnesota: Siemens Trio; Mississippi: Siemens Skyra) using a standardized protocol [5]. All MRI images were analyzed at a centralized location, namely the Mayo Clinic in Minnesota. The FreeSurfer imaging analysis software (version 5.1) was used to derive the regional grey matter volumes (including hippocampal volumes) and the estimated total intracranial volumes from the sagittal T1-weighted 3-D volumetric magnetization-prepared rapid gradient-echo (MPRAGE; 1.2 mm slices) sequences. For this project, the total grey volume was derived as the sum of the four lobar volumes (frontal, parietal, temporal, and occipital) and the deep grey subcortical structure volumes (e.g. thalamus, caudate, putamen, globus pallidum) [6]. The total brain volume was estimated from the MPRAGE sequences using in-house methods [7,8]. The white matter hyperintensities volume was quantified from the axial T2 fluid-attenuated inversion recovery (FLAIR; 5mm slices) images using a semi-automated algorithm developed by the Mayo Aging and Dementia Imaging Research Laboratory [9]. WMH were detected in every participant.

**Analytic Sample**

**
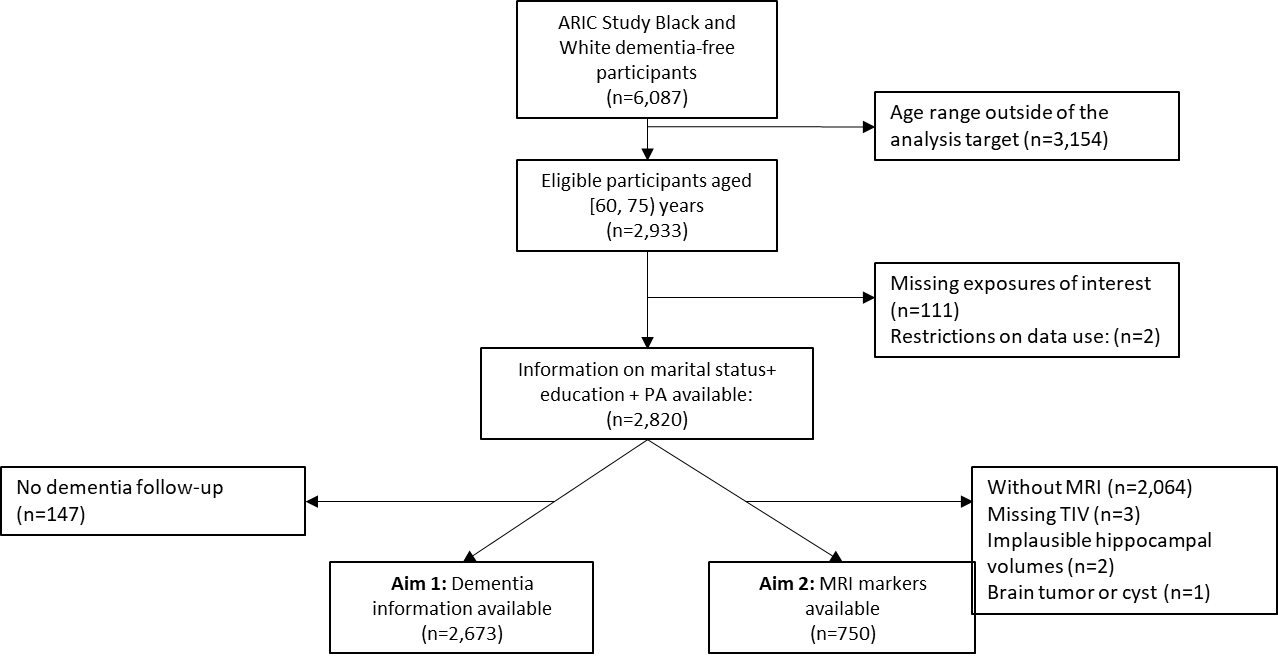
**6,538 participants were seen at the fifth clinic visit. We excluded participants with race classified as neither black or white (N=18), MRI images acquired at the wrong field center (N=28), prevalent dementia or missing dementia status at baseline (N=405), ages ≥75 years (N=3,154), restrictions on data use (N=2), missing values of marriage, physical activity, or education (N=111), leaving 2,820 participants. For the analyses of incident dementia, an additional 147 participants were removed due to a lack of follow-up or dementia diagnosis, producing 2,673 as the final analytic sample. For the analyses of brain MRI outcomes, we excluded participants with missing brain MRI (N=2,064), missing estimated total intracranial volumes (N=3), implausible hippocampal volumes (N=2), or a history of brain tumor or cyst (N=1), yielding 750 participants in the final analytic sample.

**Funding**

The Atherosclerosis Risk in Communities Study is carried out as a collaborative study supported by National Heart, Lung, and Blood Institute contracts (75N92022D00001, 75N92022D00002, 75N92022D00003, 75N92022D00004, 75N92022D00005). The ARIC Neurocognitive Study is supported by U01HL096812, U01HL096814, U01HL096899, U01HL096902, and U01HL096917 from the NIH (NHLBI, NINDS, NIA and NIDCD). The authors thank the staff and participants of the ARIC study for their important contributions. Jeannette Simino is partially funded by National Institute of General Medical Sciences grant 1P20GM144041 which established the Molecular Center of Health and Disease at the University of Mississippi Medical Center.

**References**

1 The Atherosclerosis Risk in Communities (ARIC) Study: design and objectives. The ARIC investigators. Am J Epidemiol. 1989 Apr;129(4):687–702.

2 Knopman DS, Gottesman RF, Sharrett AR, Wruck LM, Windham BG, Coker L, et al. Mild Cognitive Impairment and Dementia Prevalence: The Atherosclerosis Risk in Communities Neurocognitive Study (ARIC-NCS). Alzheimers Dement (Amst). 2016;2:1–11.

5 Schneider ALC, Selvin E, Sharrett AR, Griswold M, Coresh J, Jack CR, et al. Diabetes, Prediabetes, and Brain Volumes and Subclinical Cerebrovascular Disease on MRI: The Atherosclerosis Risk in Communities Neurocognitive Study (ARIC-NCS). Diabetes Care. 2017 Nov;40(11):1514–21.

6 Graff-Radford J, Simino J, Kantarci K, Mosley TH, Griswold ME, Windham BG, et al. Neuroimaging Correlates of Cerebral Microbleeds: The ARIC Study (Atherosclerosis Risk in Communities). Stroke. 2017 Nov;48(11):2964–72.

7 Knopman DS, Griswold ME, Lirette ST, Gottesman RF, Kantarci K, Sharrett AR, et al. Vascular imaging abnormalities and cognition: mediation by cortical volume in nondemented individuals: atherosclerosis risk in communities-neurocognitive study. Stroke. 2015 Feb;46(2):433–40.

8 Jack CR, Wiste HJ, Weigand SD, Rocca WA, Knopman DS, Mielke MM, et al. Age-specific population frequencies of cerebral β-amyloidosis and neurodegeneration among people with normal cognitive function aged 50-89 years: a cross-sectional study. Lancet Neurol. 2014 Oct;13(10):997–1005.

9 Raz L, Jayachandran M, Tosakulwong N, Lesnick TG, Wille SM, Murphy MC, et al. Thrombogenic microvesicles and white matter hyperintensities in postmenopausal women. Neurology. 2013 Mar;80(10):911–8.

10. Folsom AR, Arnett DK, Hutchinson RG, Liao F, Clegg LX, Cooper LS. Physical activity and incidence of coronary heart disease in middle-aged women and men. Med Sci Sports Exerc. 1997 Jul;29(7):901-

11. Martinez-Amezcua P, Garcia Morales E, Gabriel KP, Dooley EE, Hornikel B, Coresh J, Lin FR, Pankow JS, Sharrett AR, Schrack JA, Sullivan KJ, Reed N, Deal JA, Palta P. The Association Between Midlife Leisure-Time Physical Activity and Hearing Loss in Late Life in the Atherosclerosis Risk in Communities Study. J Gerontol A Biol Sci Med Sci. 2023 Jul 8;78(7):1292-1299.

12. Ainsworth BE, Haskell WL, Herrmann SD, Meckes N, Bassett DR Jr, Tudor-Locke C, Greer JL, Vezina J, Whitt-Glover MC, Leon AS. 2011 Compendium of Physical Activities: a second update of codes and MET values. Med Sci Sports Exerc. 2011 Aug;43(8):1575-81. doi: 10.1249/MSS.0b013e31821ece12. PMID: 21681120.

13. Palta P, Heiss G, Sharrett AR, Gabriel KP, Walker K, Evenson KR, Knopman D, Mosley TH, Wong DF, Gottesman RF. Mid- and Late-Life Leisure-Time Physical Activity and Global Brain Amyloid Burden: The Atherosclerosis Risk in Communities (ARIC)-PET Study. J Alzheimers Dis. 2020;76(1):139-147.

14. Sonsin-Diaz N, Gottesman RF, Fracica E, Walston J, Windham BG, Knopman DS, Walker KA. Chronic Systemic Inflammation Is Associated With Symptoms of Late-Life Depression: The ARIC Study. Am J Geriatr Psychiatry. 2020 Jan;28(1):87-98. doi: 10.1016/j.jagp.2019.05.011. Epub 2019 May 21. PMID: 31182350; PMCID: PMC6868307.

15. Kohout FJ, Berkman LF, Evans DA, Cornoni-Huntley J. Two shorter forms of the CES-D (Center for Epidemiological Studies Depression) depression symptoms index. J Aging Health. 1993 May;5(2):179-93. doi: 10.1177/089826439300500202. PMID: 10125443.

16. Choi NG, Ha JH. Relationship between spouse/partner support and depressive symptoms in older adults: gender difference. Aging Ment Health. 2011 Apr;15(3):307-17. doi: 10.1080/13607863.2010.513042. Epub 2010 Dec 6. PMID: 21140305; PMCID: PMC3608851.

17. Johansen, M.C., et al., *Risk of Dementia Associated With Atrial Cardiopathy: The ARIC Study.* J Am Heart Assoc, 2022. **11**(16): p. e025646.

18. Knopman, D.S., et al., *Mild Cognitive Impairment and Dementia Prevalence: The Atherosclerosis Risk in Communities Neurocognitive Study (ARIC-NCS).* Alzheimers Dement (Amst), 2016. **2**: p. 1-11.

19. McKhann, G.M., et al., *The diagnosis of dementia due to Alzheimer's disease: recommendations from the National Institute on Aging-Alzheimer's Association workgroups on diagnostic guidelines for Alzheimer's disease.* Alzheimers Dement, 2011. **7**(3): p. 263-9.

**Cardiovascular Health Study (CHS)**

**General Information**

The Cardiovascular Health Study is a population-based cohort study of risk factors for coronary heart disease and stroke in adults ≥ 65 years conducted across four field centers (1). The original predominantly European ancestry cohort of 5,201 persons was recruited in 1989-1990 from random samples of people on Medicare eligibility lists; subsequently in 1992-1993, an additional predominantly African-American cohort of 687 persons was enrolled for a total sample of 5,888. The baseline examinations consisted of a home interview and a clinic examination that assessed not only traditional risk factors but also measures of subclinical disease, including carotid ultrasound, echocardiography, electrocardiography, and pulmonary function. The study conducted extensive annual clinical exams between 1989-1999 along with semi-annual phone calls and events adjudication.

**Data Collection**

The participant characteristic data used in these analyses were derived from the baseline exam (1989-90 for the original cohort; or 1992-3 for the supplemental cohort). Cranial MRIs data is from the initial scans conducted from 1991-4.

Diabetes was defined by the use of insulin or oral hypoglycemics or fasting glucose ≥ 126 mg/dL. Hypertension was defined by an average seated blood pressure of ≥ 140 mmHg or diastolic ≥ 90 or a self-reported history of hypertension and the use of an anti-hypertensive medication. Current smoking was based on self-report. Depressive symptomatology was assessed with the Center for Epidemiological Studies-Depression (CESD) scale, using scores of > 16 as indicators of a clinically relevant level of depressive symptoms.

Local institutional review boards approved the study, and all participants provided informed consent.

*Social/Lifestyle factors –* Marital status, educational attainment, and physical activity were based on self-administered questionnaires. Physical activity was defined as > 3.75 MET-hours/week based on the total calculated kcal per week of physical activity (excluding chores).

*Dementia Ascertainment –* The CHS Cognition Study adjudicated incident cases of dementia occurring within CHS from 1992–93 to 1998–99. (2) The cohort included all 3,608 CHS study participants who had a cranial MRI scan and Modified Mini-Mental State Examination in 1991–1994. A standardized protocol was administered across the four sites to classify participants as having prevalent dementia at the time of the MRI examination or incident dementia or MCI from the time of the MRI to the end of the follow-up period (1998–1999), death, or loss to follow-up. Dementia was defined as a progressive or static deficit in at least two cognitive domains that did not necessarily include memory and was of sufficient severity to affect the participants' daily activities combined with a history of normal intellectual function. Dementia type was classified as probable or possible Alzheimer disease (AD) (National Institute of Neurological and Communicative Diseases and Stroke–Alzheimer’s Disease and Related Disorders Association criteria), probable or possible vascular dementia (State of California Alzheimer’s Disease Diagnostic and Treatment Centers criteria), mixed dementia, or other. MRI findings were used to aid in classification of dementia type but not in the initial dementia diagnosis. All dementia cases were reviewed by an adjudication committee composed of expert neurologists and psychiatrists.

Detailed methods, and baseline and follow-up results have been published (3). Following the 1998–1999 visit, participants at the Pittsburgh field center continued to be followed prospectively for incident dementia through 2013 in the Pittsburgh CHS Cognition Study (4).

*Magnetic Resonance Imaging –* After conducting an MRI pilot study, which included 303 participants between 1991 and 1992, the remaining participants who had follow-up clinic visits scheduled were invited to have cranial MRI scans during 1993 and 1994. In total, 3660 participants without contraindication who consented underwent imaging in a standard fashion (5). Magnetic resonance imaging was performed on General Electric or Picker 1.5-T scanners at 3 field centers and on a 0.35-T Toshiba scanner at the fourth. Both ARIC and CHS used the same protocols for scanning and for interpretation (6). Scanning protocol included sagittal T1-weighted localizer images and axial T1, spin-density, and T2-weighted images. Axial images had 5-mm thickness without interslice gaps.(6) Without knowledge of any clinical information, neuroradiologists at the reading center estimated white matter, ventricular, and sulcal grades using a 10-point system, from 0 to 9 (most abnormal), using a library of templates.(7) One point was added to white matter grade score for all participants to allow for the log-transformation of white matter grade score of 0.

**Analytic Sample**

Lifestyle factors and brain MRI variables were available for 2427 participants, the analytic sample was limited to the **1840** who were aged 60-74 years at the time of measurement. For the dementia aims, the analytic sample was limited to **2448** participants whose lifestyle factors were assessed prior to age 75 and who were subsequently assessed for incident dementia (those with prevalent dementia were excluded).


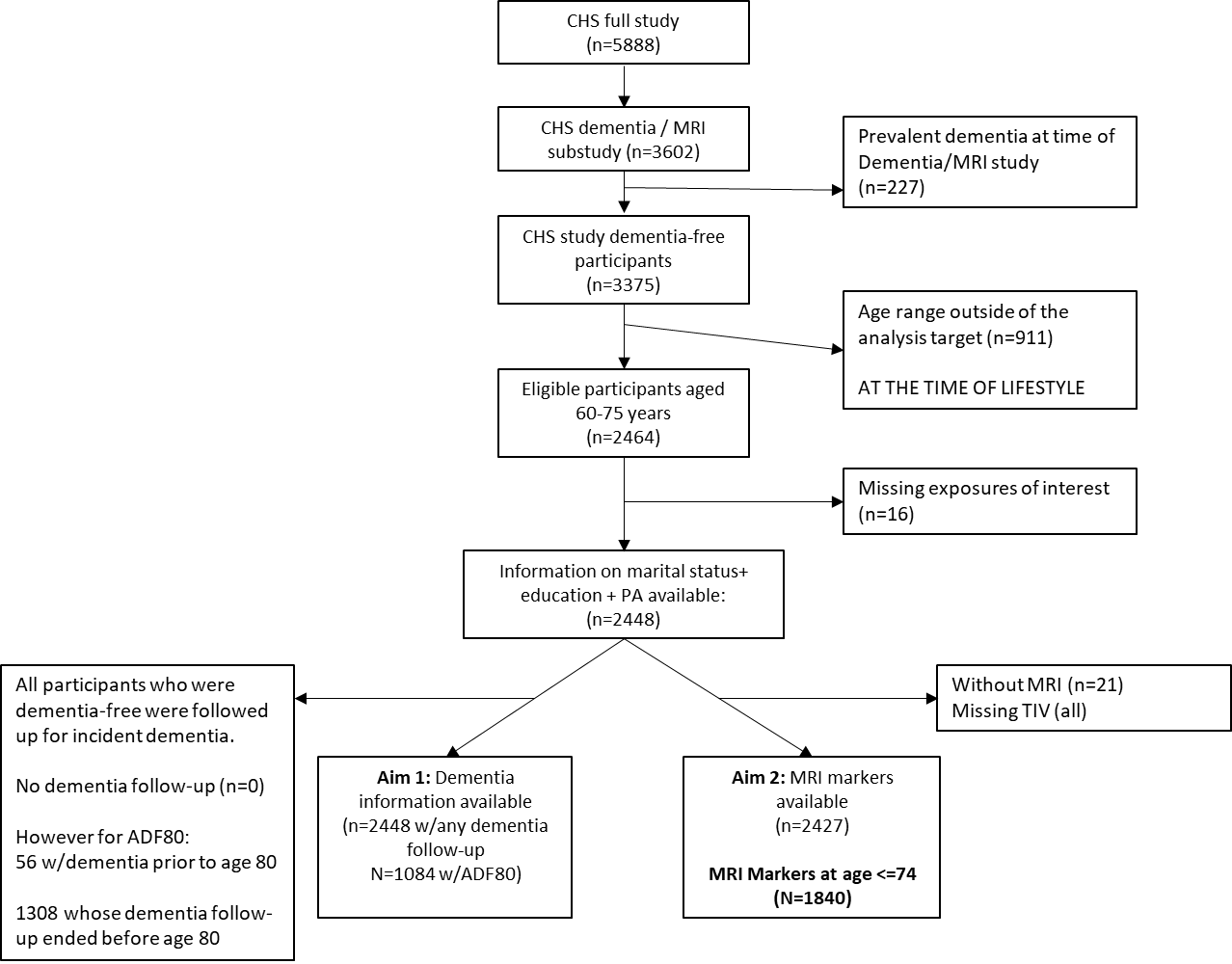


**Funding**

This research was supported by contracts HHSN268201200036C, HHSN268200800007C, HHSN268201800001C, N01HC55222, N01HC85079, N01HC85080, N01HC85081, N01HC85082, N01HC85083, N01HC85086, 75N92021D00006, N01HC15103, and grants U01HL080295, U01HL130114, and R01HL105756 from the National Heart, Lung, and Blood Institute (NHLBI), with additional contribution from the National Institute of Neurological Disorders and Stroke (NINDS). Additional support was provided by R01AG023629 and R01AG15928, R01AG20098, R01AG033193 from the National Institute on Aging (NIA). A full list of principal CHS investigators and institutions can be found at CHS-NHLBI.

**References**

1. Fried LP, Borhani NO, Enright P, Furberg CD, Gardin JM, Kronmal RA, et al. The Cardiovascular Health Study: design and rationale. Ann Epidemiol. 1991 Feb;1(3):263–76.

2. Lopez OL, Kuller LH, Fitzpatrick A, Ives D, Becker JT, Beauchamp N. Evaluation of dementia in the cardiovascular health cognition study. Neuroepidemiology. 2003 Jan-Feb;22(1):1-12.

3. Fitzpatrick AL, Kuller LH, Ives DG, Lopez OL, Jagust W, Breitner JC, Jones B, Lyketsos C, Dulberg C. Incidence and prevalence of dementia in the Cardiovascular Health Study. J Am Geriatr Soc. 2004 Feb;52(2):195-204.

4. Kuller LH, Lopez OL, Becker JT, Chang Y, Newman AB. Risk of dementia and death in the long-term follow-up of the Pittsburgh Cardiovascular Health Study-Cognition Study. Alzheimers Dement. 2016 Feb;12(2):170-183.

5. Bryan RN, Wells SW, Miller TJ, Elster AD, Jungreis CA, Poirier VC, Lind BK, Manolio TA. Infarctlike lesions in the brain: prevalence and anatomic characteristics at MR imaging of the elderly--data from the Cardiovascular Health Study. Radiology. 1997 Jan;202(1):47-54.

6. Bryan RN, Manolio TA, Schertz LD, Jungreis C, Poirier VC, Elster AD, Kronmal RA. A method for using MR to evaluate the effects of cardiovascular disease on the brain: the cardiovascular health study. AJNR Am J Neuroradiol. 1994 Oct;15(9):1625-33.

7. Longstreth WT Jr, Manolio TA, Arnold A, Burke GL, Bryan N, Jungreis CA, Enright PL, O'Leary D, Fried L. Clinical correlates of white matter findings on cranial magnetic resonance imaging of 3301 elderly people. The Cardiovascular Health Study. Stroke. 1996 Aug;27(8):1274-82.

**The Framingham Heart Study (FHS)**

**General Information**

The FHS is a single-site, community-based, ongoing cohort study that was initiated in 1948 to investigate risk factors for cardiovascular disease^1^. The Original cohort enrolled 5,209 men and women who comprised two-thirds of the adult population largely of European descent then residing in Framingham, MA. In 1971, children of the Original cohort and their spouses were invited to participate; the Offspring cohort comprises 5,124 persons (including 3,514 biological offspring)^2^ All participants gave written informed consent. The study was approved by an ethics committee and complies with the declaration of Helsinki.

**Data Collection**

FHS participants in the Offspring cohort have clinic examinations approximately once every 4 years. These include self-report medical history, lifestyle, dietary, and physical activity questionnaires, and phlebotomy, physical exam, ECG, blood biomarkers measurement and urinalysis. All participants are under continuous surveillance for CVD events, dementia, and death. Participants were invited to undergo brain MRI starting in 1999 following each of their 7^th^ (1998-2001), 8^th^ (2005-2008), and 9^th^ (2011-2014) examinations.

*Social/Lifestyle factors –* At each of the 7^th^, 8^th^, and 9^th^ examinations, participants reported on their marital status and physical activity. A physical activity index composite score was created as previously described^3^; participants with physical activity index scores in the lowest 25% were considered physically inactive and the other participants were considered physically active. Educational attainment was determined based on self-report at the time of MRI.

*Dementia Ascertainment –* Dementia surveillance at FHS has been previously described.^4^ Participants in the offspring cohort have undergone cognitive monitoring since 1987, first with a subjective memory questionnaire at their 4^th^ examination (1987-1991), then with Mini-Mental State Examination (MMSE) assessments at each examination since their 5^th^ examination (1991-1995), and finally with 45-minute comprehensive neuropsychological testing every 5-6 years since 1999 [18]. . A decline in MMSE scores; flagging by the neuropsychological assessment, subjective cognitive decline reported by the participant or a family member, either spontaneously between examinations or during annual health-status updates; referral by a treating physician or by ancillary investigators of the Framingham Heart Study; or after review of outside medical records will trigger thorough assessment including neurologic and neuropsychological examinations. Participants suspected of having dementia are reviewed by a dementia review panel consisting of at least one neurologist and one neuropsychologist. All cases of possible cognitive decline and dementia documented in the Framingham Heart Study have been reviewed by the panel. Cases detected before 2001 were given a repeat review after 2001 in order to apply up-to-date diagnostic criteria. The dementia review panel determines whether a person has dementia, as well as the dementia subtype and the date of diagnosis, using serial neurologic and neuropsychological assessments, telephone interviews with caregivers, medical records, neuroimaging results, and, when applicable, autopsy data. Dementia diagnoses are made according to the *Diagnostic and Statistical Manual of Mental Disorders*, fourth edition (DSM-IV).

*Magnetic Resonance Imaging* – The MRI protocol in FHS has been described previously^5^. Briefly, participants were imaged by a variety of MRI machines varying in field strength from 1 to 3 Tesla. Two sequences were used: a 3-dimensional T1-weighted and 2 or 3-dimensional FLAIR imaging. All images were transferred to and processed by the University of California Davis Medical Center without knowledge of clinical information. Segmentation and quantification of brain volume measures were performed by automated procedures with quality control. Total cerebral cranial volume (TCV) was determined using a convolutional neural network method^6^. Images were further segmented into 4 tissue types (gray, white, CSF, and WMH volumes) using previously published methods(6-8)^9^. Hippocampal analyses were performed using atlas based^10^ diffeomorphic approach^11^ with the minor modification of label refinement. Non-linear co-registration of images to the DKT atlas^12^ enabled calculation of regional gray matter volumes(9, 10). MR infarcts were identified according to standard protocols(11) with excellent reliability(12). Participants without WMH (WMHV equal to 0) were excluded.

**Analytic Sample**

Of 5,124 participants in the Offspring cohort, 2,671 attended at least one of their 7^th^, 8^th^, and 9^th^ examinations aged between 60 and 74 years, with information on the lifestyle exposures of interest. For those who attended more than one such examination, we selected the examination attended at the age closest to 67.5 as the baseline examination.

Aim 1 Sample: Of the 2,671 participants, 1,089 underwent brain MRI as a call back after the clinic exam. Of those, 13 were missing TIV and, at the time of the MRI, 5 were missing information on prevalent dementia, 10 had prevalent dementia, 8 were missing information on other neurological conditions and 35 had other prevalent neurological conditions, yielding a sample of n=1,511.

Aim 2 Sample: Of the 2,671 participants, at baseline, 22 were missing information on prevalent dementia, 9 had prevalent dementia, and 298 did not have follow-up for incident dementia, yielding a sample of n=2,342.


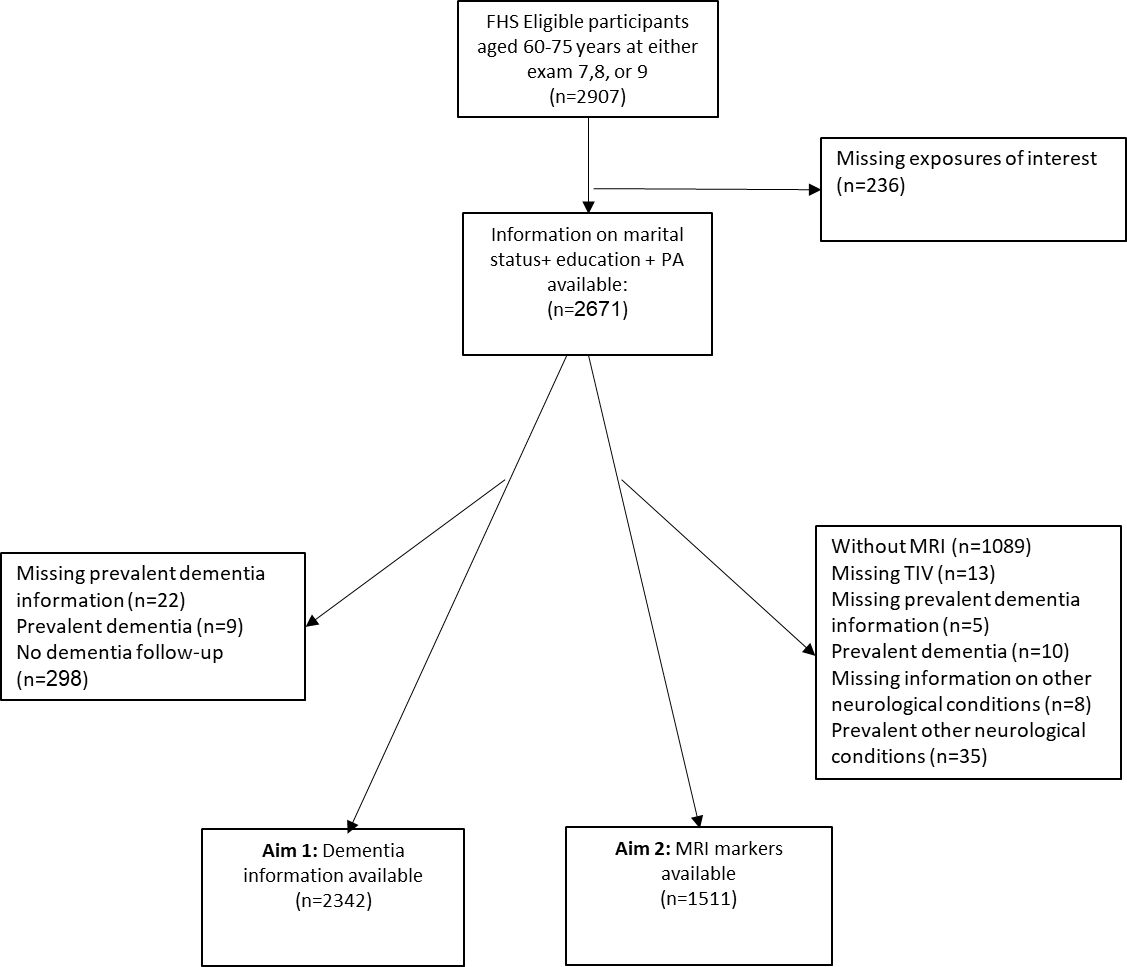


**Funding**

This study was supported by grants from the National Heart, Lung, and Blood Institute contract for the Framingham Heart Study (contract No. N01-HC-25195, No. HHSN268201500001I, and No. 75N92019D00031), the National Institute on Aging (R01 AG054076, R01 AG049607, U01 AG052409, R01 AG059421, RF1 AG063507, RF1 AG066524, U01 AG058589) and the National Institute of Neurological Disorders and Stroke (R01 NS017950 and UH2 NS100605).

Dr. Satizabal receives support from the Texas Alzheimer’s Research and Care Consortium (2020-58-81-CR) and NIH (R01 AG059727, UF1 NS125513, and P30 AG066546). Drs. Seshadri and Satizabal receive support from P30AG066546 (South Texas Alzheimer’s Disease Research Center).

**References**

1 Dawber TR, Kannel WB. The Framingham study. An epidemiological approach to coronary heart disease. Circulation. 1966 Oct;34(4):553–5.

2 Feinleib M, Kannel WB, Garrison RJ, McNamara PM, Castelli WP. The Framingham Offspring Study. Design and preliminary data. Prev Med. 1975 Dec;4(4):518–25.

3. Kannel WB. Some health benefits of physical activity. The Framingham Study. Archives of Internal Medicine. 1979 Aug 1;139(8):857–61.

4. Satizabal CL, Beiser AS, Chouraki V, Chêne G, Dufouil C, Seshadri S. Incidence of Dementia over Three Decades in the Framingham Heart Study. N Engl J Med. 2016 Feb 11;374(6):523-32. doi: 10.1056/NEJMoa1504327. PMID: 26863354; PMCID: PMC4943081.

5. Massaro JM, D'Agostino RB, Sr., Sullivan LM, et al. Managing and analysing data from a large-scale study on Framingham Offspring relating brain structure to cognitive function. Stat Med 2004;23:351-367.

6. Fletcher E, DeCarli C, Fan AP, Knaack A. Convolutional Neural Net Learning Can Achieve Production-Level Brain Segmentation in Structural Magnetic Resonance Imaging. Front Neurosci 2021;15:683426.

7. Fletcher E, Singh B, Harvey D, Carmichael O, Decarli C. Adaptive image segmentation for robust measurement of longitudinal brain tissue change. Conf Proc IEEE Eng Med Biol Soc 2012;2012:5319-5322.

8. Maillard P, Lu H, Arfanakis K, et al. Instrumental validation of free water, peak-width of skeletonized mean diffusivity, and white matter hyperintensities: MarkVCID neuroimaging kits. Alzheimers Dement (Amst) 2022;14:e12261.

9. Fletcher E, Carmichael O, Decarli C. MRI non-uniformity correction through interleaved bias estimation and B-spline deformation with a template. Conf Proc IEEE Eng Med Biol Soc 2012;2012:106-109.

10. Bocchetta M, Boccardi M, Ganzola R, et al. Harmonized benchmark labels of the hippocampus on magnetic resonance: The EADC-ADNI project. Alzheimers Dement 2014.

11. Rueckert D, Aljabar P, Heckemann RA, Hajnal JV, Hammers A. Diffeomorphic registration using B-splines. Med Image Comput Comput Assist Interv 2006;9:702-709.

12. Desikan RS, Segonne F, Fischl B, et al. An automated labeling system for subdividing the human cerebral cortex on MRI scans into gyral based regions of interest. Neuroimage 2006;31:968-980.

13. Aljabar P, Heckemann RA, Hammers A, Hajnal JV, Rueckert D. Multi-atlas based segmentation of brain images: atlas selection and its effect on accuracy. Neuroimage 2009;46:726-738.

14. Wardlaw JM, Smith EE, Biessels GJ, et al. Neuroimaging standards for research into small vessel disease and its contribution to ageing and neurodegeneration. Lancet Neurol 2013;12:822-838.

15. DeCarli C, Massaro J, Harvey D, et al. Measures of brain morphology and infarction in the framingham heart study: establishing what is normal. Neurobiol Aging 2005;26:491-510.

**The Rotterdam Study (RS)**

**General Information**

The Rotterdam Study is a large, prospective, population-based cohort of the Netherlands including community-dwelling adults from Ommoord (a suburb of Rotterdam). A comprehensive description of the study design has been published previously.[1] In summary, all inhabitants of Ommoord aged ≥55 years were invited to participate in 1990, from which 7983 individuals enrolled (RS-I). Subsequently, the cohort was expanded thrice: first in 2000, which resulted in the inclusion of 3,011 individuals who had reached the eligible age or had moved into the study area (RS-II), later in 2006, with 3,932 individuals aged 45 or over (RS-III) and recently in 2015 with 3,005 individuals aged 40 or over (RS-IV). Participants take part in comprehensive interviews and visit the research facility for an in-person examination every 3-6 years. In addition, participants are monitored continuously through electronic linkage of medical records with the study database.

The Rotterdam Study has been approved by the Medical Ethics Committee of the Erasmus University Medical Center (registration number MEC 02.1015), in accordance with the Population Screening Act, which is executed by the Dutch Ministry of Health, Welfare and Sport (license number 1071272-159531-PG).

**Data Collection**

Medical history and socio-demographic variables were assessed by standardized questionnaires during home interview. Blood samples, and measurement of body height were carried out during routine examinations at the research center. Type 2 diabetes was defined as a fasting serum glucose level ≥7.0 mmol/L (126 mg/dL), or a nonfasting serum glucose level ≥11.1 mmol/L (200 mg/dL), and/or the use of blood glucose-lowering medication. MRI of the brain is part of the routine examination since 2005.

*Social/Lifestyle factors –* Information on educational attainment and marital status were obtained during home interview. Physical activity was enquired during the same interview, using the LASA Physical Activity Questionnaire (LAPAQ). The questionnaire has previously been validated, with a reasonably well test-retest reliability. [2] The questionnaire includes questions regarding the frequency and duration of walking, cycling, sports, gardening and housework performed per week in the past two weeks. In addition, there were two questions regarding sports that were not captured by the previous questions. If specific questions were missing, they were imputed by using age- and sex-specific means. To quantify intensity of the activities, we used metabolic equivalent of task (MET) for all activities, according to the updated Compendium of Physical Activities of 2011. If participants engaged in sports that were not in this compendium (e.g. ‘treadmill’, ‘indoor sports’, ‘power plate’), no MET-values were assigned. Physical activity was assessed using the LASA Physical Activity Questionnaire (LAPAQ), and expressed in METhours/week. For this analysis, we used the variables that indicated moderate to vigorous activity, which was converted to METminutes/week.

*Dementia Ascertainment –* Participants were screened for dementia at baseline and at follow-up examinations using a three-step protocol.[3] Screening was done using the Mini-Mental State Examination (MMSE) and the Geriatric Mental Schedule (GMS) organic level. Screen-positives (MMSE <26 or GMS organic level >0) subsequently underwent an examination and informant interview with the Cambridge Examination for Mental Disorders in the Elderly. Participants who were suspected of having dementia underwent extra neuropsychological testing if necessary. Additionally, for persons not visiting the research center, the total cohort was continuously monitored for dementia through computerized linkage of the study database and digitized medical records from general practitioners and the Regional Institute for Outpatient Mental Health Care. When information on neuroimaging was required and available, it was used for decision making on the diagnosis. Ultimately, a consensus panel, led by a neurologist, decided on the final diagnosis in accordance with standard criteria for dementia (DSM-III-R). Follow-up for dementia was complete for 99.5 % of potential person-years in the original cohort and for 98.5 % of potential person-years in the extended cohort.

*Magnetic Resonance Imaging –* MRI of the brain was performed on a 1.5T scanner (General Electric Healthcare, Milwaukee, WI) using an 8-channel head coil. Imaging acquisition included a high-resolution axial T1-weighted sequence, a fluid-attenuated inversion recovery sequence, a proton density–weighted sequence, and a T2*-weighted gradient echo sequence. Details about the sequences, preprocessing, and the classification algorithm have been described previously [4]. Total intracranial and parenchymal volumes and volume of white matter hyperintensities (WMHs) were quantified via automated tissue segmentation [5]. These segmentations were visually inspected and manually corrected if needed. Segmentation of the hippocampus was performed using FreeSurfer 6.1 [6]. Participants without WMH (WMHV equal to 0) were excluded.

**Analytic Sample**


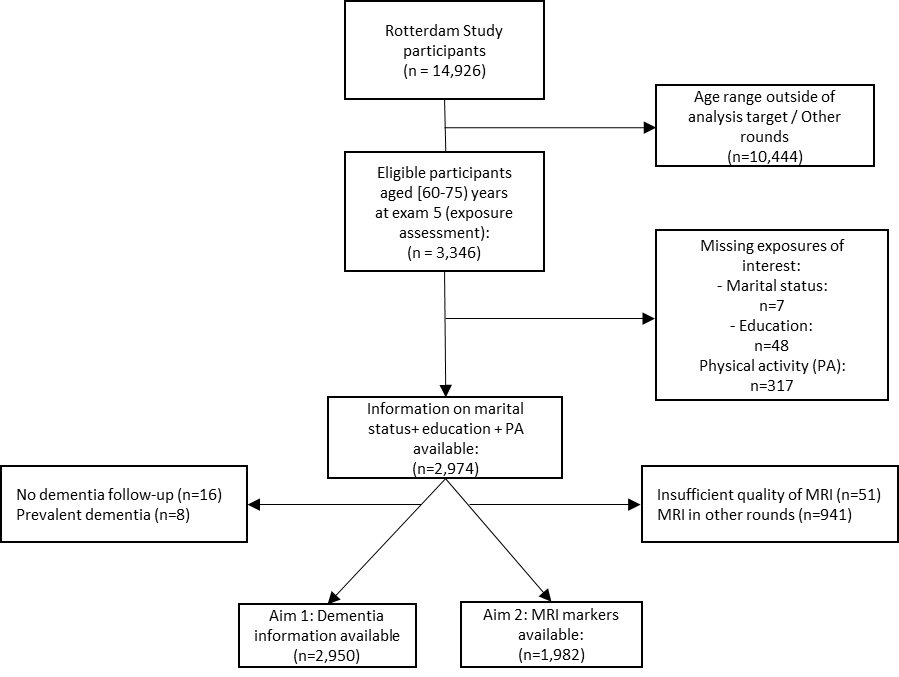
Of 14,926 participants of the Rotterdam Study, we included participants from the fifth visit of the first cohort (RS-I-5), the third visit of the second cohort (RS-II-3) and the second visit of the third cohort (RS-III-2), who were between 60-<75 years old at time of exposure assessment (n=3,346, see Flowchart). Among these participants, 7 participants had missing information on marital status, 48 had missing information on education and 317 had missing data on physical activity, yielding a study sample of 2,974 participants with complete data on all exposures of interest. For aim 1, we excluded 8 participants with prevalent dementia and 16 participants with no information on cognitive status, leading to an analytic sample of 2,950 study participants. For aim 2, we excluded those with insufficient quality of brain MRI and participants who only had MRI in other rounds (n=941). This led to a total sample of 1,982 participants with data on exposures of interest and brain MRI.

**Funding**

The Rotterdam Study is supported by the Erasmus MC University Medical Center and Erasmus University Rotterdam, the Netherlands Organization for Scientific Research (NWO), the Netherlands Organization for Health Research and Development (ZonMW), the Research Institute for Diseases in the Elderly (RIDE), the Ministry of Education, Culture and Science, the Ministry of Health, Welfare and Sport, The European Commission (DGXII), the Netherlands Genomics Initiative (NGI), and the Municipality of Rotterdam. This study was partly performed as part of the Netherlands Consortium of Dementia Cohorts (NCDC), which receives funding in the context of Deltaplan Dementie from ZonMW Memorabel (projectnr 73305095005) and Alzheimer Nederland. Julia Neitzel was supported by a grant from European Union’s Horizon 2020 (MSCA-IF-GF no.101032288).

**References**

1. Ikram MA, Brusselle G, Ghanbari M, Goedegebure A, Ikram MK, Kavousi M, et al. Objectives, design and main findings until 2020 from the Rotterdam Study. Eur J Epidemiol. 2020 May;35(5):483–517.
2. Stel VS, Smit JH, Pluijm SMF, Visser M, Deeg DJH, Lips P. Comparison of the LASA Physical Activity Questionnaire with a 7-day diary and pedometer. Journal of Clinical Epidemiology. 2004 Mar;57(3):252–8.
3. de Bruijn RF, Bos MJ, Portegies ML, Hofman A, Franco OH, Koudstaal PJ, Ikram MA. The potential for prevention of dementia across two decades: the prospective, population-based Rotterdam Study. BMC Med. 2015 Jul 21;13:132. doi: 10.1186/s12916-015-0377-5.
4. Ikram MA, van der Lugt A, Niessen WJ, Koudstaal PJ, Krestin GP, Hofman A, et al. The Rotterdam Scan Study: design update 2016 and main findings. Eur J Epidemiol. 2015 Dec;30(12):1299–315.
5. de Boer R, Vrooman HA, van der Lijn F, Vernooij MW, Ikram MA, van der Lugt A, et al. White matter lesion extension to automatic brain tissue segmentation on MRI. NeuroImage. 2009 May;45(4):1151–61.
6. Schmidt MF, Storrs JM, Freeman KB, Jack CR, Turner ST, Griswold ME, et al. A comparison of manual tracing and FreeSurfer for estimating hippocampal volume over the adult lifespan. Hum Brain Mapp. 2018 Febas.;39(6):2500–13.

**The Study of Health in Pomerania (SHIP)**

**General Information**

The Study of Health in Pomerania (SHIP) is a prospective population-based cohort of adults from Western Pomerania, a north-eastern region in Germany of approximately 220,000 inhabitants (1,2). A random sample of 7,008 residents aged 20-79 years from the population of Western Pomerania was drawn for the first SHIP cohort in 1997 (SHIP-START). 4,308 subjects agreed to participate in the baseline examinations conducted between 1997 and 2001, with follow-up examinations 5 years, 11 years, 16 years, and 21 years after baseline. A separate sample of 8,016 residents aged 20–79 years was drawn for the second cohort (SHIP-TREND), and 4,420 of them agreed to participate in the baseline examinations between 2008 and 2012. For this study, data were derived from the second follow-up examinations of SHIP-START and the baseline examinations of SHIP-TREND. All participants gave written informed consent. The study was approved by the ethics committee of the University Medicine Greifswald and complies with the declaration of Helsinki.

**Data Collection**

Medical history and socio-demographic variables were assessed by standardized questionnaires during a computer-assisted face-to-face interview. Taking of blood samples as well as measurements of blood pressure (BP), body height, and weight were carried out during the subsequent medical examinations. BP was measured three times from the right brachial artery after a 10‐minute rest in a supine position and the average of the second and third measurement was considered. Hypertension was defined according ISH-WHO 1999 (1=either systolic BP ≥ 140mmHg, or diastolic BP ≥ 90mmHg, or intake of antihypertensive medications; 0=otherwise). Diabetes was defined based on self-report, intake of anti-diabetic medication (ATC code A10), glycated hemoglobin ≥ 6.5% (International Expert Committee 2009), or blood glucose levels ≥ 11.1 mmol/l (IDF-WHO 2006). APOE genotype was determined on the basis of the two single-nucleotide polymorphisms rs429358 and rs7412 (3). Depressive symptoms were assessed based on the Beck Depression Inventory (BDI)-II score in SHIP-TREND (total score >=14) and the Clinical Interview for Depression (CID) in SHIP-START ((1) feeling sad or depressed for more than two weeks during last 12 months, or (2) loss of interests, feeling tired, and lack of energy almost every day during at least two weeks during last 12 months). Magnetic resonance imaging (MRI) of the head was performed during a second visit.

*Social/lifestyle factors –* Social and lifestyle factors were assessed by standardized questionnaires. Participants were considered physically active when engaging in leisure-time physical activities for more than 1h per week.

*Dementia status –* Dementia diagnoses were extracted from health insurance records of the Association of Statutory Health Insurance Physicians of Mecklenburg-Western Pomerania (Kassenärztliche Vereinigung Mecklenburg-Vorpommern, KVMV; ICD-10 codes F00, F01, F02, F03, G30, and G31). Diagnoses until 2017 were considered in the analyses.

*Magnetic Resonance Imaging* **–** T1-weighted and fluid-attenuated inversion recovery (FLAIR) scans of the head were acquired with a 1.5 T Siemens Magnetom Avanto scanner (Siemens, Erlangen, Germany) (4). The following parameters were used: T1: orientation=axial plane, TR=1,900 ms, TE=3.37 ms, flip angle 15 °, slice thickness=1 mm, and resolution 1 mm x 1 mm, FLAIR: orientation=axial plane, TR=5,000 ms, TE=325 ms, slice thickness=3 mm, and resolution 0.9 mm × 0.9 mm. T1-weighted scans were processed with the image-processing pipeline FreeSurfer version 7.1 which is documented and freely available for download online (http://surfer.nmr.mgh.harvard.edu) (5). The processing includes segmentation of the cerebral cortex and subcortical regions as well as calculation of brain volume, gray matter volume, and total hippocampal volume (sum of left and right hippocampal volume). FreeSurfer also gives an estimate of the total intracranial volume which can be used to account for some of the variability between the study participants. After preprocessing and coregistration of T1-weighted and FLAIR scans, white matter lesions were segmented using the Brain Intensity AbNormality Classification Algorithm (BIANCA) (6). WMH were detected in every participant.

**Analytic Sample**

The data used in our analyses were derived from the second follow-up examinations of SHIP-START and the baseline examinations of SHIP-TREND, conducted between 2008 and 2012. Data on education level, marital status, and self-reported physical activity were available for 1,962 participants aged 60 to 74 years without prevalent dementia. MRI data were available for a subset of 944 study participants. Of those, 34 were excluded from the analyses because of major structural abnormalities of the brain (e.g. large cysts, tumors), cerebral ischemia, or hemorrhage.

*
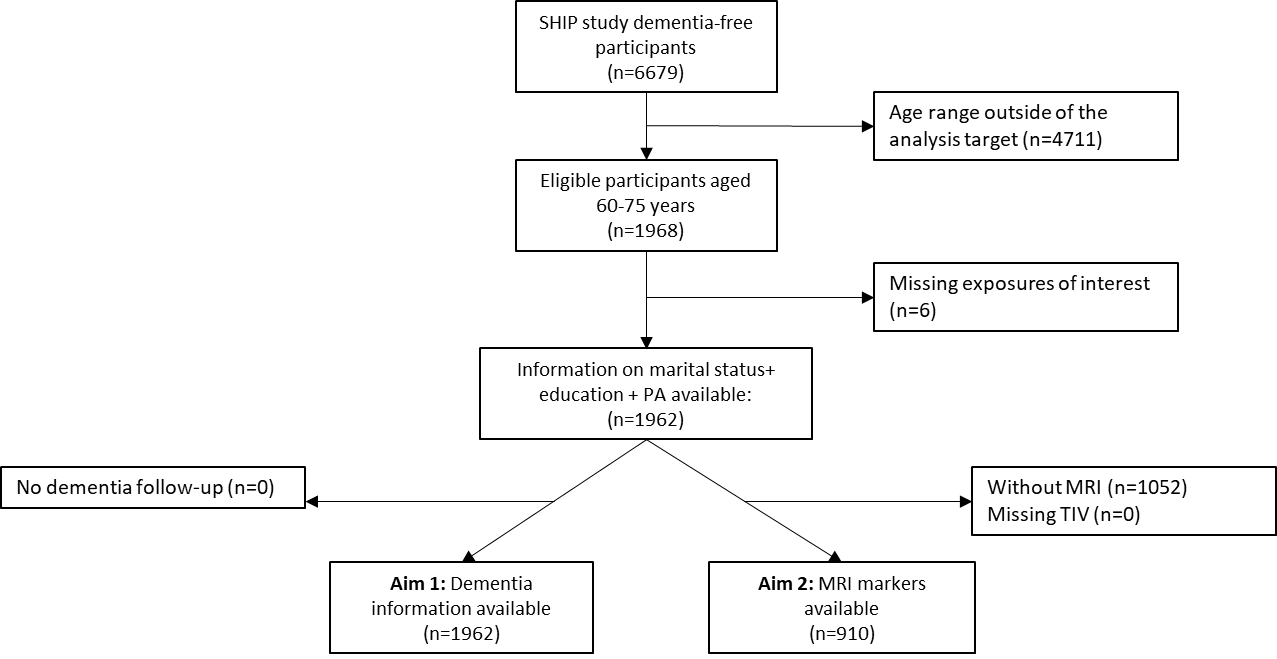
*

**Acknowledgements**

The authors are grateful to the staff and study participants of SHIP for their invaluable efforts in establishing this study.

**Funding**

The Study of Health in Pomerania (SHIP) is part of the Community Medicine Research net (CMR) (http://www.medizin.uni-greifswald.de/icm) of the University of Greifswald funded by grants from the German Federal Ministry of Education and Research (BMBF, grants 01ZZ96030 & 01ZZ0701). The MRIs in SHIP and SHIP-Trend were supported by a joint grant from Siemens Healthineers, Erlangen, Germany, and the Federal State of Mecklenburg-Western Pomerania. This study was further supported by National Institute of Health (NIH) grant AG059421.

**References**

1. Völzke H, Alte D, Schmidt CO, Radke D, Lorbeer R, Friedrich N, et al. Cohort profile: the study of health in Pomerania. Int J Epidemiol. 2011 Apr;40(2):294–307.

2. Völzke H, Schössow J, Schmidt CO, Jürgens C, Richter A, Werner A, et al. Cohort Profile Update: The Study of Health in Pomerania (SHIP). Int J Epidemiol. 2022 Mar 29;dyac034.

3. Bonk S, Kirchner K, Ameling S, Garvert L, Völzke H, Nauck M, et al. APOE ε4 in Depression-Associated Memory Impairment—Evidence from Genetic and MicroRNA Analyses. Biomedicines. 2022 Jun 30;10(7):1560.

4. Hosten N, Bülow R, Völzke H, Domin M, Schmidt CO, Teumer A, et al. SHIP-MR and Radiology: 12 Years of Whole-Body Magnetic Resonance Imaging in a Single Center. Healthcare. 2022 Jan;10(1):33.

5. Fischl B. FreeSurfer. Neuroimage. 2012 Aug 15;62(2):774–81.

6. Griffanti L, Zamboni G, Khan A, Li L, Bonifacio G, Sundaresan V, et al. BIANCA (Brain Intensity AbNormality Classification Algorithm): A new tool for automated segmentation of white matter hyperintensities. Neuroimage. 2016 Nov 1;141:191–205.

**Supplemental Methods 2: Description of sensitivity analyses**

In order to assess the robustness of our results, a series of sensitivity analyses were undertaken. First, to assess potential selection into MRI subsamples within the 3C study sample as an example, we also applied inverse probability weighting (IPW). The weight (assessing the probability of being included in the MRI analysis) of each participant was obtained by fitted values of a multivariable logistic regression modelling inclusion status (yes/no) as a dependent variable and including education level, marital status, physical activity, the covariates used in the primary analysis, as well as other covariates such as Mini Mental State Examination (MMSE) score at time of MRI. Linear regressions between each exposure of interest and MRI markers were then weighted by the inverse of the stabilized probability of remaining in the sample. Second, as RS presents a high proportion of physically active participants (almost 96%), the meta-analysis was rerun without the RS sample. Third, CHS assessed graded white matter lesions, which may lead to differences in estimates effect sizes, the meta-analysis regarding associations between factors of interest and white matter hyperintensities volume was rerun without the CHS sample. Fourth, due to potential contextual differences in education levels across studies, we evaluated whether meta-analysis results regarding education levels varied across geographical regions by conducting a meta-regression with study regions (i.e. USA (ARIC, CHS, FHS) vs European Union (3C, RS, SHIP)) as a covariate. To investigate the impact of differences in categorization of education levels on associations, which may lead to differences in associations across cohorts, we also reran the main analysis of the association between education levels and dementia risk by using different cut-offs within the 3C study sample as an example.

**Supplemental Figure 1:** Directed Acyclic Graphs (DAGs) of the associations between education level, marital status, or physical activity and brain health outcomes.

**
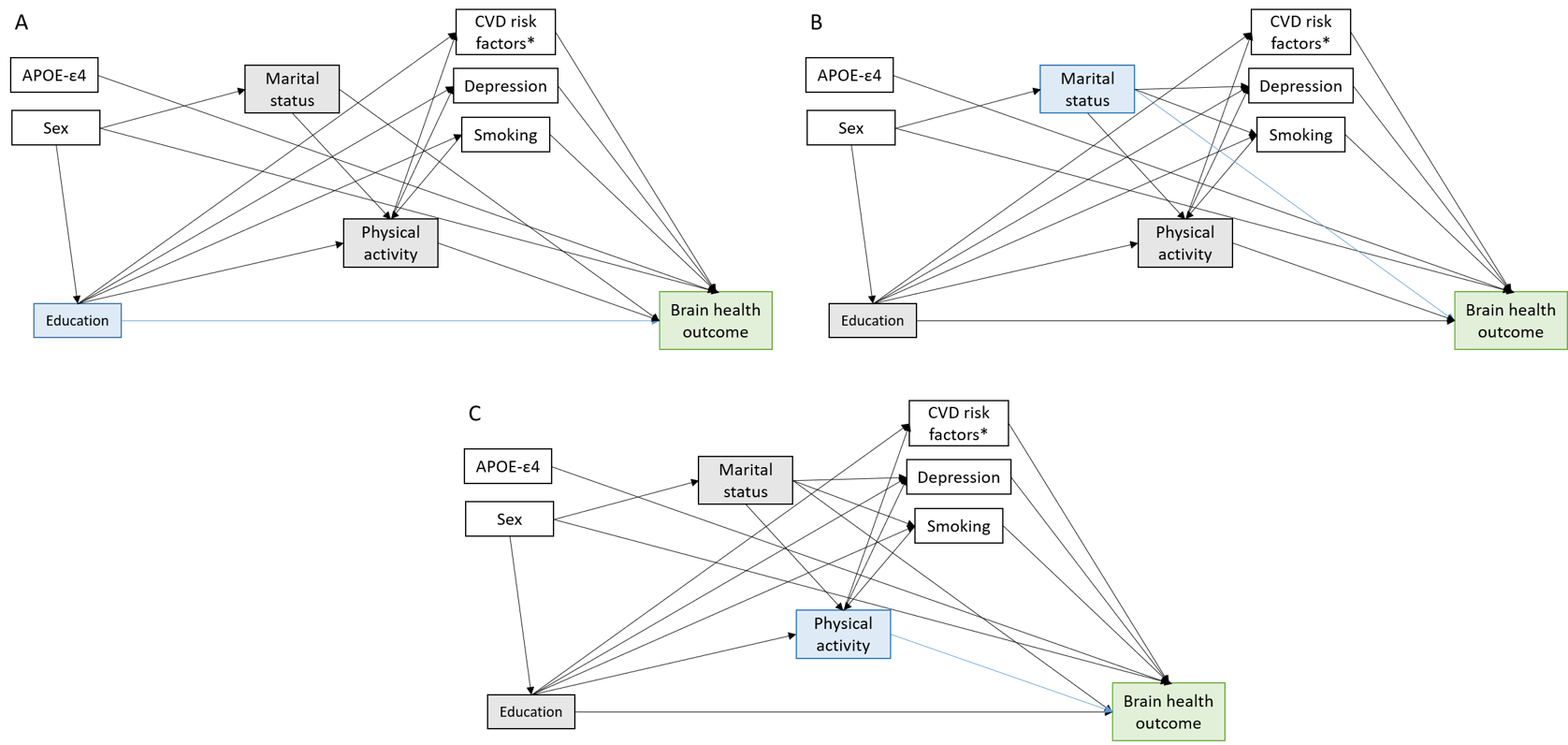
**

A: DAG for education level as the main exposure. B: DAG for marital status as the main exposure. C: DAG for physical activity as the main exposure.

* CVD risk factors: hypertension, diabetes, and BMI.

**Supplemental Table 1:** Overall associations between education, marital status, physical activity and dementia risk across models.

| Exposures | Model | Number of  Studies | Overall Effect (95% CI) | Heterogeneity Test | |  |
| --- | --- | --- | --- | --- | --- | --- |
|  |  |  |  | *Q* | *P*-value | *I²* |
|  | Model 1 (main) | 6 | 0.78 (0.63 ; 0.96) | 4.77 | 0.44 | 0.0 |
| **Intermediate education level** | Model 2 | 6 | 0.76 (0.63 ; 0.91) | 3.68 | 0.60 | 0.0 |
|  | Model 3 | 6 | 0.79 (0.63 ; 0.98) | 5.17 | 0.40 | 3.3 |
|  | Model 4 | 6 | 0.78 (0.64 ; 0.96) | 4.50 | 0.48 | 0.0 |
|  | Model 5 | 6 | 0.78 (0.63 ; 0.96) | 4.60 | 0.47 | 0.0 |
|  | Model 1 (main) | 6 | 0.65 (0.59 ; 0.72) | 1.23 | 0.94 | 0.0 |
| **High education level** | Model 2 | 6 | 0.65 (0.59 ; 0.71) | 1.04 | 0.96 | 0.0 |
|  | Model 3 | 6 | 0.67 (0.64 ; 0.70) | 0.22 | 1.00 | 0.0 |
|  | Model 4 | 6 | 0.68 (0.66 ; 0.71) | 0.20 | 1.00 | 0.0 |
|  | Model 5 | 6 | 0.68 (0.66 ; 0.70) | 0.09 | 1.00 | 0.0 |
|  | Model 2 (main) | 6 | 0.88 (0.72 ; 1.07) | 7.79 | 0.17 | 35.8 |
| **Marital status** | Model 3 | 6 | 0.88 (0.73 ; 1.05) | 6.83 | 0.23 | 26.8 |
|  | Model 4 | 6 | 0.89 (0.74 ; 1.07) | 6.85 | 0.23 | 27.0 |
|  | Model 5 | 6 | 0.94 (0.76 ; 1.16) | 9.31 | 0.10 | 46.3 |
| **Physical activity** | Model 3 (main) | 6 | 0.73 (0.52 ; 1.04) | 23.2 | 0.0003 | 78.4 |
|  | Model 4 | 6 | 0.75 (0.53 ; 1.06) | 22.32 | 0.0005 | 77.6 |
|  | Model 5 | 6 | 0.76 (0.57 ; 1.03) | 13.27 | 0.02 | 62.3 |

Abbreviation: TBV: total brain volume, GMV: total gray matter volume, HV: hippocampal volume, WMHV: white matter hyperintensities volume.

Intermediate and high education vs. low education, married or in a partnership vs. currently unmarried, physically active vs. inactive.

Brain volumes unit is cm^3^ and WMHV were log-transformed.

Model 2 were adjusted for age, sex, education level, marital status, ethnicity (when applicable), and other cohort specific covariates. Model 3 = model 2 + physical activity. Model 4 = model 3 + smoking status. Model 5 = model 4 + APOE-ε4 status, hypertension, diabetes, BMI, and high depressive symptoms.

Heterogeneity measures: Cochran’s Q statistic and associated P-value, I²: percentage of variation across studies that is due to heterogeneity rather than chance.

**Supplemental Table 2:** Meta-analyzed associations between physical activity (vs. inactivity) and dementia and MRI outcomes after exclusion of the Rotterdam Study.

| Outcomes | Number of  Studies | Overall Effect (95% CI) | Heterogeneity Test | |  |
| --- | --- | --- | --- | --- | --- |
|  |  |  | *Q* | *P*-value | *I²* |
| Dementia risk | 5 | 0.701 (0.472 ; 1.042) | 21.58 | 0.0002 | 0.82 |
| Total brain volume | 4 | 5.169 (1.578 ; 8.760) | 3.25 | 0.355 | 0.08 |
| Total gray matter volume | 4 | 3.583 (0.098 ; 7.068) | 5.62 | 0.132 | 0.47 |
| Hippocampal volume | 4 | 0.031 (-0.060 ; 0.123) | 5.50 | 0.139 | 0.46 |
| White matter hyperintensities volume | 5 | -0.086 (-0.177 ; 0.006) | 8.73 | 0.068 | 0.54 |
| Models were adjusted for age, sex, education level, marital status, ethnicity (when applicable), and other cohort specific covariates. Heterogeneity measures: Cochran’s Q statistic and associated P-value, I²: percentage of variation across studies that is due to heterogeneity rather than chance. | | | | | |

**Supplemental Table 3:** Geographical differences in the associations of education levels and outcomes of interest

|  | USA | EU | β (95% CI) | *P value* |
| --- | --- | --- | --- | --- |
| Dementia risk |  |  |  |  |
| Intermediate education (EU vs USA) | 0.818 (0.629 ; 1.065) | 0.740 (0.327 ; 1.675) | -0.127 (-0.624 ; 0.369) | 0.516 |
| High education (EU vs USA) | 0.651 (0.468 ; 0.906) | 0.649 (0.602 ; 0.699) | -0.004 (-0.264 ; 0.256) | 0.969 |
| Total brain volume |  |  |  |  |
| Intermediate education (EU vs USA) | 2.664 (-5.239 ; 10.566) | 0.090 (-8.578 ; 8.758) | -2.641 (-13.235 ; 7.954) | 0.486 |
| High education (EU vs USA) | 2.622 (-18.760 ; 24.003) | 2.735 (-6.510 ; 11.979) | 0.221 (-11.089 ; 11.531) | 0.954 |
| Total gray matter volume |  |  |  |  |
| Intermediate education (EU vs USA) | -1.896 (-23.468 ; 19.694) | 0.706 (-3.967 ; 5.380) | 2.602 (-4.144 ; 9.349) | 0.307 |
| High education (EU vs USA) | 0.752 (-36.629 ; 38.134) | 3.730 (-2.900 ; 10.359) | 2.924 (-6.604 ; 12.451) | 0.401 |
| Hippocampal volume |  |  |  |  |
| Intermediate education (EU vs USA) | 0.053 (-0.258 ; 0.364) | -0.036 (-0.174 ; 0.101) | -0.091 (-0.295 ; 0.113) | 0.251 |
| High education (EU vs USA) | 0.111 (-0.269 ; 0.491) | 0.021 (-0.117 ; 0.159) | -0.089 (-0.288 ; 0.110) | 0.251 |
| White matter hyperintensities volume |  |  |  |  |
| Intermediate education (EU vs USA) | -0.020 (-0.119 ; 0.079) | -0.019 (-0.097 ; 0.060) | 0.001 (-0.087 ; 0.089) | 0.966 |
| High education (EU vs USA) | -0.046 (-0.124 ; 0.032) | 0.023 (-0.045 ; 0.092) | 0.068 (-0.004 ; 0.142) | 0.058 |
| β estimates represent the differences between EU and USA studies in the associations of education levels and outcomes of interest using meta regression.  Intermediate and high education vs. low education.  Models were adjusted for age, sex, ethnicity (when applicable), and other cohort specific covariates. | | | | |

**Supplemental Table 4:** Associations between different definition cut-offs of education level and dementia risk in the 3C study

|  | OR (95% CI) |
| --- | --- |
| Primary education definition |  |
| Low (No education, primary education, short secondary school, and short vocational school) | Ref |
| Intermediate (high school and intermediate vocational school) | 0.64 (0.47 – 0.90) |
| High (more than high school and high vocational school) | 0.68 (0.49 – 0.93) |
| Alternate definition 1 |  |
| Low (No education and primary education) | Ref |
| Intermediate (short secondary school, short vocational school, high school and intermediate vocational school) | 0.52 (0.40 – 0.67) |
| High (more than high school and high vocational school) | 0.49 (0.35 – 0.69) |
| Alternate definition 2 |  |
| Low (No education and primary education) | Ref |
| Intermediate (short secondary school and short vocational school) | 0.54 (0.41 – 0.72) |
| High (high school and intermediate vocational school, more than high school and high vocational school) | 0.48 (0.36 – 0.63) |
| Alternate definition 3 |  |
| Low (No education, primary education, short secondary school, short vocational school, high school and intermediate vocational school) | Ref |
| High (more than high school and high vocational school) | 0.75 (0.55 – 1.02) |
| Alternate definition 4 |  |
| Low (No education and primary education) | Ref |
| High (short secondary school and short vocational school, high school and intermediate vocational school, more than high school and high vocational school) | 0.51 (0.40 – 0.65) |
| Alternate definition 5 |  |
| Low (No education, primary education, short secondary school, and short vocational school) | Ref |
| High (high school and intermediate vocational school, more than high school and high vocational school) | 0.66 (0.52 – 0.85) |
| Cox models including education, marital status, physical activity, sex, and study site. | |

**Supplemental Table 5:** Demographics and characteristics of samples with information available on social and lifestyle factors and MRI markers (n=8517)

|  | **ARIC** | **CHS** | **FHS** | **RS** | **SHIP** | **3C** |
| --- | --- | --- | --- | --- | --- | --- |
| **N** | 750 | 1840 | 1511 | 1982 | 910 | 1524 |
| **Demographics and Risk Factors** |  |  |  |  |  |  |
| Mean age at lifestyle factors, y | 71.6 (2.0) | 68.5 (1.9) | 67.6 (3.2) | 67.7 (4.1) | 66.3 (4.1) | 70.1 (2.4) |
| Male | 285 (38.0) | 732 (39.8) | 716 (47.4) | 893 (45.3) | 428 (47) | 634 (41.6) |
| APOE-ε4 carriers | 220 (30.3) | 456 (24.8) | 345 (24.0) | 518 (27.8) | 209 (24.2) | 348 (22.8) |
| Education level  < High school  High school/GED  More than high school | 75 (10.0)  242 (32.3)  433 (57.7) | 410 (22.3)  560 (30.4)  870 (47.3) | 56 (3.7)  409 (27.1)  1046 (69.2) | 125 (6.3) 1351 (68.2)  506 (25.5) | 324 (35.6)  346 (38.0)  240 (26.4) | 900 (59.1)  290 (19.0)  334 (21.9) |
| Marital status  Married / in a partnership  Currently unmarried | 488 (65.1)  262 (34.9) | 1335 (72.6)  505 (27.4) | 1119 (74.1)  392 (25.9) | 1527 (77.0)  455 (23.0) | 757 (83.2)  153 (16.8) | 973 (63.8)  551 (36.2) |
| Physically active | 473 (63.1) | 1420 (77.2) | 1176 (77.8) | 1516 (76.5) | 536 (58.9) | 835 (54.8) |
| Current smoking | 62 (8.3) | 228 (12.4) | 110 (7.3) | 197 (10.0) | 72 (7.9) | 100 (6.6) |
| Diabetes | 234 (31.5) | 264 (14.3) | 197 (13.0) | 297 (15.1) | 137 (15.1) | 121 (7.9) |
| Hypertension | 531 (71.1) | 994 (54.0) | 858 (56.8) | 1406 (70.9) | 609 (66.9) | 1100 (72.2) |
| BMI | 29.6 (5.8) | 27.2 (4.6) | 28.2 (5.0) | 27.3 (4.0) | 28.7 (4.2) | 25.7 (3.9) |
| High depressive symptoms | 49 (6.5) | 65 (3.5) | 110 (8.0) | 111 (5.8) | 80 (8.9) | 263 (17.3) |
| **MRI Measures** |  |  |  |  |  |  |
| Delay between exposures assessment and MRI* | 312 [137 , 492] | 1109 [920 , 1221] | 239 [74 , 551] | 31 [16 , 58] | 17 [5 , 36] | 165 [66 , 253] |
| Total intracranial volume, cm^3^ | 1369.1 (149.5) | - | 1267 (131) | 1139.1 (112.4) | 1560.9 (160.8) | 1326.7 (154.7) |
| Total brain volume, cm^3^ | 1033.3 (107.1) | - | 962 (101) | 931.9 (89.2) | 959.2 (94.8) | 982.2 (96.8) |
| Total gray matter volume, cm^3^ | 449.9 (45.2) | - | 518 (50) | 527.9 (51.3) | 492.2 (45) | 510.3 (50.3) |
| Total hippocampal volume, cm^3^ | 7.3 (0.8) | - | 6.6 (0.7) | 6.7 (0.7) | 7.6 (0.8) | 6.9 (0.8) |
| White matter hyperintensities volume  Continuous cm^3^, median [IQR]  Grade, median [IQR] | 8.4 [4.6 , 15.3]  - | -  2 [1 , 2] | 1.03 [0.55, 1.88]  - | 3.5 [0.2 , 103.5]  - | 0.51 [0.2 , 1.59]  - | 3.9 [2.7 , 5.8]  - |
| Data are numbers (percentage) unless otherwise indicated. *: median number of days [IQR]  Abbreviations: ARIC: Atherosclerosis Risk in Communities, CHS: Cardiovascular Health Study, FHS: Framingham Heart Study, RS: Rotterdam Study, SHIP: Study of Health in Pomerania, 3C: Three-City, APOE: apolipoprotein E, GED: General Education Development, BMI: Body mass index, SD: Standard Deviation  US cohorts: ARIC, CHS, FHS ; European cohorts: RS, SHIP, 3C.  Currently unmarried includes widowed, divorced, separated, or single. | | | | | | |

**Supplemental Table 6:** Overall associations between education, marital status, physical activity and brain MRI markers across models.

| Outcomes | Exposures | Models | Number of  Studies | Overall Effect (95% CI) | Heterogeneity Test | |  |
| --- | --- | --- | --- | --- | --- | --- | --- |
|  |  |  |  |  | *Q* | *P*-value | *I²* |
| **Total brain volume** | **Intermediate education level** | Model 2 | 5 | 0.676 (-3.162 ; 4.515) | 3.63 | 0.459 | 0.0 |
|  |  | Model 3 | 5 | 0.441 (-3.417 ; 4.300) | 3.70 | 0.448 | 0.0 |
|  |  | Model 4 | 5 | 0.519 (-3.348 ; 4.385) | 3.72 | 0.445 | 0.0 |
|  |  | Model 5 | 5 | 0.261 (-3.880 ; 4.403) | 4.00 | 0.406 | 0.0 |
|  | **High education level** | Model 2 | 5 | 2.975 (-0.684 ; 6.634) | 3.13 | 0.537 | 0.0 |
|  |  | Model 3 | 5 | 2.646 (-1.107 ; 6.400) | 3.28 | 0.512 | 0.0 |
|  |  | Model 4 | 5 | 2.684 (-1.259 ; 6.627) | 3.58 | 0.466 | 0.0 |
|  |  | Model 5 | 5 | 2.108 (-3.391 ; 7.608) | 6.17 | 0.187 | 35.1 |
|  | **Marital status** | Model 3 | 5 | 2.291 (0.291 ; 4.290) | 2.30 | 0.680 | 0.0 |
|  |  | Model 4 | 5 | 2.123 (0.113 ; 4.132) | 2.31 | 0.680 | 0.0 |
|  |  | Model 5 | 5 | 2.268 (1.316 ; 3.220) | 0.48 | 0.975 | 0.0 |
|  | **Physical activity** | Model 4 | 5 | 4.916 (2.744 ; 7.088) | 2.91 | 0.573 | 0.0 |
|  |  | Model 5 | 5 | 4.021 (1.445 ; 6.597) | 3.94 | 0.414 | 0.0 |
| **Gray matter volume** | **Intermediate education level** | Model 2 | 5 | 0.087 (-2.516 ; 2.690) | 3.16 | 0.532 | 0.0 |
|  |  | Model 3 | 5 | -0.147 (-2.833 ; 2.539) | 3.35 | 0.501 | 0.0 |
|  |  | Model 4 | 5 | -0.100 (-2.864 ; 2.665) | 3.54 | 0.471 | 0.0 |
|  |  | Model 5 | 5 | -0.333 (-2.959 ; 2.292) | 3.06 | 0.547 | 0.0 |
|  | **High education level** | Model 2 | 5 | 2.664 (-1.151 ; 6.479) | 6.91 | 0.141 | 42.1 |
|  |  | Model 3 | 5 | 2.330 (-1.325 ; 5.985) | 6.49 | 0.166 | 38.3 |
|  |  | Model 4 | 5 | 2.224 (-1.556 ; 6.003) | 6.98 | 0.137 | 42.7 |
|  |  | Model 5 | 5 | 2.188 (-2.062 ; 6.438) | 7.81 | 0.099 | 48.8 |
|  | **Marital status** | Model 3 | 5 | 1.804 (0.103 ; 3.505) | 2.95 | 0.566 | 0.0 |
|  |  | Model 4 | 5 | 1.636 (-0.216 ; 3.487) | 3.46 | 0.484 | 0.0 |
|  |  | Model 5 | 5 | 1.431 (-0.273 ; 3.134) | 2.05 | 0.726 | 0.0 |
|  | **Physical activity** | Model 4 | 5 | 3.085 (0.399 ; 5.771) | 7.06 | 0.133 | 43.3 |
|  |  | Model 5 | 5 | 2.518 (0.939 ; 4.097) | 2.55 | 0.636 | 0.0 |

Abbreviation: TBV: total brain volume, GMV: total gray matter volume, HV: hippocampal volume, WMHV: white matter hyperintensities volume.

Intermediate and high education vs. low education, married or in a partnership vs. currently unmarried, physically active vs. inactive.

Brain volumes unit is cm^3^ and WMHV were log-transformed.

Model 2 were adjusted for age, sex, education level, marital status, ethnicity (when applicable), and other cohort specific covariates. Model 3 = model 2 + physical activity. Model 4 = model 3 + smoking status. Model 5 = model 4 + APOE-ε4 status, hypertension, diabetes, BMI, and high depressive symptoms.

Heterogeneity measures: Cochran’s Q statistic and associated P-value, I²: percentage of variation across studies that is due to heterogeneity rather than chance.

**Supplemental Table 6 continued:** Overall associations between education, marital status, physical activity and brain MRI markers across models.

| Outcomes | Exposures | Models | Number of  Studies | Overall Effect (95% CI) | Heterogeneity Test | |  |
| --- | --- | --- | --- | --- | --- | --- | --- |
|  |  |  |  |  | *Q* | *P*-value | *I²* |
| **Hippocampal volume** | **Intermediate education level** | Model 2 | 5 | -0.020 (-0.096 ; 0.057) | 4.81 | 0.307 | 16.8 |
|  |  | Model 3 | 5 | -0.021 (-0.096 ; 0.053) | 4.52 | 0.340 | 11.6 |
|  |  | Model 4 | 5 | -0.021 (-0.097 ; 0.055) | 4.81 | 0.307 | 16.9 |
|  |  | Model 5 | 5 | -0.023 (-0.105 ; 0.058) | 5.07 | 0.280 | 21.2 |
|  | **High education level** | Model 2 | 5 | 0.039 (-0.036 ; 0.115) | 4.56 | 0.335 | 12.3 |
|  |  | Model 3 | 5 | 0.035 (-0.039 ; 0.109) | 4.35 | 0.361 | 8.1 |
|  |  | Model 4 | 5 | 0.035 (-0.038 ; 0.109) | 4.30 | 0.367 | 6.9 |
|  |  | Model 5 | 5 | 0.038 (-0.043 ; 0.119) | 4.75 | 0.314 | 15.7 |
|  | **Marital status** | Model 3 | 5 | 0.035 (0.012 ; 0.058) | 0.98 | 0.912 | 0.0 |
|  |  | Model 4 | 5 | 0.034 (0.007 ; 0.061) | 1.40 | 0.844 | 0.0 |
|  |  | Model 5 | 5 | 0.030 (0.007 ; 0.053) | 0.88 | 0.928 | 0.0 |
|  | **Physical activity** | Model 4 | 5 | 0.024 (-0.026 ; 0.073) | 5.07 | 0.280 | 21.2 |
|  |  | Model 5 | 5 | 0.020 (-0.024 ; 0.063) | 3.52 | 0.475 | 0.0 |
| **White matter hyperintensity volume** | **Intermediate education level** | Model 2 | 6 | -0.019 (-0.053 ; 0.015) | 1.96 | 0.855 | 0.0 |
|  |  | Model 3 | 6 | -0.014 (-0.046 ; 0.018) | 1.83 | 0.872 | 0.0 |
|  |  | Model 4 | 6 | -0.015 (-0.048 ; 0.019) | 1.87 | 0.867 | 0.0 |
|  |  | Model 5 | 6 | -0.012 (-0.038 ; 0.014) | 1.22 | 0.943 | 0.0 |
|  | **High education level** | Model 2 | 6 | -0.021 (-0.067 ; 0.025) | 3.57 | 0.614 | 0.0 |
|  |  | Model 3 | 6 | -0.012 (-0.057 ; 0.033) | 3.27 | 0.658 | 0.0 |
|  |  | Model 4 | 6 | -0.011 (-0.050 ; 0.029) | 2.45 | 0.784 | 0.0 |
|  |  | Model 5 | 6 | 0.001 (-0.057 ; 0.060) | 4.81 | 0.440 | 0.0 |
|  | **Marital status** | Model 3 | 6 | -0.038 (-0.105 ; 0.029) | 7.88 | 0.163 | 36.5 |
|  |  | Model 4 | 6 | -0.027 (-0.080 ; 0.027) | 5.45 | 0.364 | 8.2 |
|  |  | Model 5 | 6 | -0.034 (-0.095 ; 0.028) | 6.98 | 0.222 | 28.4 |
|  | **Physical activity** | Model 4 | 6 | -0.055 (-0.101 ; -0.009) | 4.79 | 0.442 | 0.0 |
|  |  | Model 5 | 6 | -0.065 (-0.116 ; -0.015) | 5.43 | 0.366 | 7.9 |

Abbreviation: TBV: total brain volume, GMV: total gray matter volume, HV: hippocampal volume, WMHV: white matter hyperintensities volume.

Intermediate and high education vs. low education, married or in a partnership vs. currently unmarried, physically active vs. inactive.

Brain volumes unit is cm^3^ and WMHV were log-transformed.

Model 2 were adjusted for age, sex, education level, marital status, ethnicity (when applicable), and other cohort specific covariates. Model 3 = model 2 + physical activity. Model 4 = model 3 + smoking status. Model 5 = model 4 + APOE-ε4 status, hypertension, diabetes, BMI, and high depressive symptoms.

Heterogeneity measures: Cochran’s Q statistic and associated P-value, I²: percentage of variation across studies that is due to heterogeneity rather than chance.

**Supplemental Table 7:** Associations between exposures of interest and brain MRI markers, in the original and weighted models, in the 3C study.

| Outcome | Exposure | Model | Effect (95% CI) | *P*-value |
| --- | --- | --- | --- | --- |
| **TBV** | Intermediate education | Main | -3.437 (-8.247 ; 1.373) | 0.162 |
|  |  | Weighted | -4.456 (-9.262 ; 0.351) | 0.069 |
|  | High education | Main | 5.010 (0.341 ; 9.679) | 0.036 |
|  |  | Weighted | 4.082 (-0.828 ; 8.992) | 0.103 |
|  | Married | Main | 2.888 (-1.175 ; 6.951) | 0.164 |
|  |  | Weighted | 3.146 (-0.941 ; 7.233) | 0.131 |
|  | Physically active | Main | 2.763 (-1.000 ; 6.526) | 0.150 |
|  |  | Weighted | 3.175 (-0.595 ; 6.944) | 0.099 |
| **GMV** | Intermediate education | Main | -1.353 (-4.863 ; 2.157) | 0.450 |
|  |  | Weighted | -1.586 (-5.143 ; 1.972) | 0.382 |
|  | High education | Main | 5.291 (1.885 ; 8.697) | 0.002 |
|  |  | Weighted | 5.068 (1.430 ; 8.706) | 0.006 |
|  | Married | Main | 1.681 (-1.284 ; 4.646) | 0.267 |
|  |  | Weighted | 2.276 (-0.750 ; 5.303) | 0.140 |
|  | Physically active | Main | 1.986 (-0.760 ; 4.732) | 0.157 |
|  |  | Weighted | 2.360 (-0.431 ; 5.151) | 0.097 |
| **HV** | Intermediate education | Main | -0.064 (-0.146 ; 0.018) | 0.133 |
|  |  | Weighted | -0.043 (-0.128 ; 0.042) | 0.322 |
|  | High education | Main | 0.060 (-0.020 ; 0.140) | 0.144 |
|  |  | Weighted | 0.056 (-0.031 ; 0.143) | 0.210 |
|  | Married | Main | 0.053 (-0.018 ; 0.124) | 0.136 |
|  |  | Weighted | 0.059 (-0.0139 ; 0.131) | 0.113 |
|  | Physically active | Main | 0.009 (-0.056 ; 0.074) | 0.776 |
|  |  | Weighted | 0.013 (-0.054 ; 0.079) | 0.711 |
| **WMHV** | Intermediate education | Main | -0.011 (-0.087 ; 0.065) | 0.787 |
|  |  | Weighted | -0.016 (-0.096 ; 0.063) | 0.686 |
|  | High education | Main | 0.054 (-0.021 ; 0.129) | 0.156 |
|  |  | Weighted | 0.062 (-0.019 ; 0.143) | 0.134 |
|  | Married | Main | -0.065 (-0.130 ; -0.000) | 0.052 |
|  |  | Weighted | -0.079 (-0.147 ; -0.012) | 0.021 |
|  | Physically active | Main | -0.057 (-0.118 ; 0.004) | 0.067 |
|  |  | Weighted | -0.072 (-0.135 ; -0.010) | 0.023 |
| Abbreviation: TBV: total brain volume, GMV: total gray matter volume, HV: hippocampal volume, WMHV: white matter hyperintensities volume.  Analyses were adjusted for education, marital status, physical activity, age, delay between exposures and MRI, total intracranial volume, sex, ethnicity (when applicable), APOE-ε4 status, smoking status, hypertension, diabetes, BMI, high depressive symptoms, and other cohort specific covariates.  Intermediate and high education vs. low education, married vs. currently unmarried, physically active vs. inactive.  Brain volumes unit is cm3 and WMHV were log-transformed. | | | | |

**Supplemental Table 8:** Meta-analyzed associations between exposures of interest and white matter hyperintensities volume after excluding the Cardiovascular Health Study

| Outcomes | Exposures | Number of  Studies | Overall Effect (95% CI) | Heterogeneity Test | |  |
| --- | --- | --- | --- | --- | --- | --- |
|  |  |  |  | *Q* | *P*-value | *I²* |
| **WMHV** | Intermediate education | 5 | -0.013 (-0.078 ; 0.052) | 2.05 | 0.726 | 0.0 |
|  | High education | 5 | 0.006 (-0.070 ; 0.081) | 2.72 | 0.606 | 0.0 |
|  | Married | 5 | -0.046 (-0.140 ; 0.047) | 6.58 | 0.160 | 39.2 |
|  | Physically active | 5 | -0.098 (-0.163 ; -0.034) | 4.19 | 0.381 | 4.5 |

Intermediate and high education vs. low education, married vs. currently unmarried, physically active vs. inactive.

Brain volumes unit is cm3 and WMHV were log-transformed.

Models for education were adjusted for age, sex, ethnicity (when applicable), and other cohort specific covariates. Models for marital status were adjusted for age, sex, education level, ethnicity (when applicable), and other cohort specific covariates. Models for physical activity were adjusted for age, sex, education level, marital status, ethnicity (when applicable), and other cohort specific covariates.

Heterogeneity measures: Cochran’s Q statistic and associated P-value, I²: percentage of variation across studies that is due to heterogeneity rather than chance.
